# Quantifying the impact of COVID-19 control measures using a Bayesian model of physical distancing

**DOI:** 10.1371/journal.pcbi.1008274

**Published:** 2020-12-03

**Authors:** Sean C. Anderson, Andrew M. Edwards, Madi Yerlanov, Nicola Mulberry, Jessica E. Stockdale, Sarafa A. Iyaniwura, Rebeca C. Falcao, Michael C. Otterstatter, Michael A. Irvine, Naveed Z. Janjua, Daniel Coombs, Caroline Colijn

**Affiliations:** 1 Pacific Biological Station, Fisheries and Oceans Canada, Nanaimo, Canada; 2 Department of Biology, University of Victoria, Victoria, Canada; 3 Department of Mathematics, Simon Fraser University, Burnaby, Canada; 4 Department of Mathematics and Institute of Applied Mathematics, University of British Columbia, Vancouver, Canada; 5 British Columbia Centre for Disease Control, Vancouver, Canada; 6 School of Population and Public Health, University of British Columbia, Vancouver, Canada; 7 British Columbia Children’s Hospital Research Institute, Vancouver, Canada; University of Illinois at Urbana-Champaign, UNITED STATES

## Abstract

Extensive non-pharmaceutical and physical distancing measures are currently the primary interventions against coronavirus disease 2019 (COVID-19) worldwide. It is therefore urgent to estimate the impact such measures are having. We introduce a Bayesian epidemiological model in which a proportion of individuals are willing and able to participate in distancing, with the timing of distancing measures informed by survey data on attitudes to distancing and COVID-19. We fit our model to reported COVID-19 cases in British Columbia (BC), Canada, and five other jurisdictions, using an observation model that accounts for both underestimation and the delay between symptom onset and reporting. We estimated the impact that physical distancing (social distancing) has had on the contact rate and examined the projected impact of relaxing distancing measures. We found that, as of April 11 2020, distancing had a strong impact in BC, consistent with declines in reported cases and in hospitalization and intensive care unit numbers; individuals practising physical distancing experienced approximately 0.22 (0.11–0.34 90% CI [credible interval]) of their normal contact rate. The threshold above which prevalence was expected to grow was 0.55. We define the “contact ratio” to be the ratio of the estimated contact rate to the threshold rate at which cases are expected to grow; we estimated this contact ratio to be 0.40 (0.19–0.60) in BC. We developed an R package ‘covidseir’ to make our model available, and used it to quantify the impact of distancing in five additional jurisdictions. As of May 7, 2020, we estimated that New Zealand was well below its threshold value (contact ratio of 0.22 [0.11–0.34]), New York (0.60 [0.43–0.74]), Washington (0.84 [0.79–0.90]) and Florida (0.86 [0.76–0.96]) were progressively closer to theirs yet still below, but California (1.15 [1.07–1.23]) was above its threshold overall, with cases still rising. Accordingly, we found that BC, New Zealand, and New York may have had more room to relax distancing measures than the other jurisdictions, though this would need to be done cautiously and with total case volumes in mind. Our projections indicate that intermittent distancing measures—if sufficiently strong and robustly followed—could control COVID-19 transmission. This approach provides a useful tool for jurisdictions to monitor and assess current levels of distancing relative to their threshold, which will continue to be essential through subsequent waves of this pandemic.

## Introduction

Coronavirus disease 2019 (COVID-19), caused by the severe acute respiratory syndrome coronavirus 2 (SARS-CoV-2), has now spread worldwide and resulted in over 20 million diagnosed cases and 700,000 confirmed deaths globally as of August 11, 2020 [1]. Estimates of COVID-19 case fatality rates from Hubei, China, the rest of China, and other countries range from 0.3% to above 5% in different populations at various times [2, 3], with an estimate of 1.4% [4] currently favoured in some analyses [5]. Age [6] and comorbidities (hypertension, cardiovascular and respiratory disease [7]) are strong risk factors for severe illness, hospitalization, and death. Furthermore, COVID-19 poses severe challenges for health care, with risks that requirements will exceed hospital bed, critical care, and ICU capacities even in well-resourced health care systems. In the current absence of a vaccine or effective therapeutic options, widespread non-pharmaceutical interventions including testing, contact tracing, isolation and quarantine, hand hygiene, and physician distancing, along with broad physical or social distancing, are the main interventions currently available to reduce transmission. Countries have used a variety of such physical or social distancing measures including cancelling mass gatherings, closing restaurants, work-from-home orders, and “lockdowns” of varying strictness.

**Fig 1:**
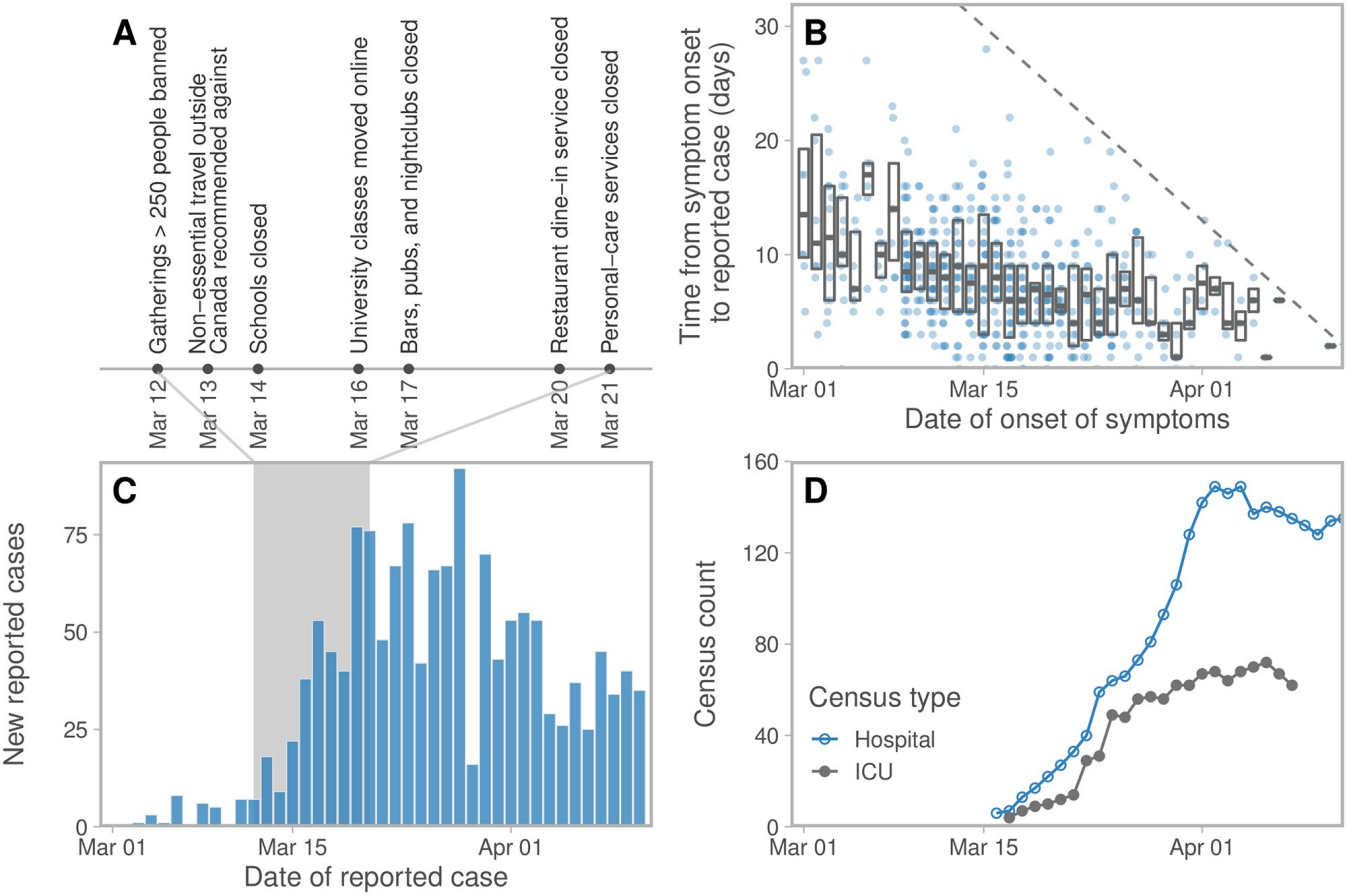
Information regarding COVID-19 in British Columbia, Canada. (A) Key physical distancing measures implemented in response to COVID-19. Schools closed for an annual two-week break on March 14 and then were declared indefinitely closed on March 17. (B) Time from symptom onset to reporting from case-specific data as of April 11, 2020 and (C) reported cases per day. The dashed line in panel B represents the line above which cases, by definition, have not been reported yet. Boxes indicate interquartile range and median values. (D) Hospitalization and ICU (Intensive Care Unit) census counts. All data are from the BC Centre for Disease Control [10].

In British Columbia (BC), Canada, for example, the first case of infection was detected on January 26, 2020 with sporadic cases related to travel until March 8, followed by a sustained increase in cases. A number of measures were implemented over the following weeks to reduce transmission (Fig 1). However, the direct impact of these measures on transmission is not known. Distancing measures have high economic, health, and social impacts. Thus, there is an urgent need to understand what level of contact rate and physical distancing measures are optimal to reduce transmission. Once initial transmission has been brought under control, as in China and Korea [8, 9] as of March/April 2020, there remains the question of what relaxation in social measures could keep transmission under control.

There have been a number of models simulating the impact of broad physical distancing measures [8, 11–13]. Direct estimates of the strength and impact of distancing measures have focused on the effective reproduction number over time, using approaches based on reported deaths [14] or confirmed cases [15]. These estimates are influenced by the assumed serial interval distribution, the infection fatality rate and the delay between symptom onset and death. In comparisons among European Union countries, estimates assume that physical-distancing measures impact each location equally and that all deaths are reported [14]. Estimates of the effective reproduction number based on reported cases have been adjusted for the delay between symptom onset and reporting [15, 16], but do not accommodate underestimation or asymptomatic or weakly symptomatic individuals. Furthermore, the effective reproduction number is a broad summary of the overall growth of the epidemic, and is not a direct estimate of the impact of physical distancing on the contact patterns relevant to transmission.

Here, we introduce an epidemiological model of physical distancing and assess the degree to which contact rates have changed—for the population that is participating in physical distancing—due to recent policy measures. We focus on BC, and also apply our methods to New York, Florida, Washington, California, and New Zealand. We quantify how close jurisdictions are to the threshold at which cases begin to rise, and we explore the impact of reducing distancing measures in BC.

## Methods

### Data

We fit the physical distancing model to case-count data from British Columbia from March 1, 2020 (when a total of eight cases had been detected in the province) to April 11, 2020 at which time 1445 cases had been confirmed. These data are available in press releases from the BC Centre for Disease Control (BCCDC) [10], from the public data dashboard [17], and from the code repository associated with this paper. Testing procedures were adapted over the course of the outbreak. In particular, lab testing criteria were changed on March 16 to focus on hospitalized patients, healthcare workers, long-term care facility residents/staff, and those people part of an existing cluster and experiencing respiratory symptoms. This led to high variability in case counts in the surrounding days with some large jumps in the number of identified cases. We accounted for this in the model by adjusting the testing fraction *ψ*_*r*_ to accommodate widening the testing pool and thereby increasing the fraction of infected individuals being tested (Table 1). There was also variability in the daily testing rate. During March, the daily number of completed tests ranged from approximately 100 to 3500, and did not strictly increase over time.

**Table 1:** Values and sources for British Columbia parameterization of the model (see Supplemental Methods and Table B in S1 Text for other jurisdictions). The duration of the infectious period is shorter than the duration of severe illness, accounting for self-isolation and less severe illnesses. The quarantine parameter *q* reflects approximately 1/5 of severe cases either ceasing to transmit due to hospitalization or completely self-isolating. The model depends on the combination *u*_*r*_/(*u*_*r*_ + *u*_*d*_), the fraction engaged in physical distancing, estimated from the survey data cited above. The testing patterns have changed over time, with laboratories increasing the numbers of tests on approximately March 14 (motivating our change in *ψ*_*r*_).

Symbol	Definition	Specified/fitted value	Justification
*N*	Population size	5,100,000	[23]
*D*	Mean duration of the infectious period	5 days	[24, 25]
*k*_1_	(time to infectiousness)^−1^ (*E*_1_ to *E*_2_)	0.2 days^−1^	[26–28]
*k*_2_	(time period of pre-symptomatic transmissibility)^−1^ (*E*_2_ to *I*)	1 days^−1^	[27, 28]
*q*	Quarantine rate	0.05	[29]
*u*_d_	Rate of people moving to physical distancing	0.1	[20]
*u*_*r*_	Rate of people returning from physical distancing	0.02	[20]
*ψ*_*r*_	Proportion of anticipated cases on day *r* that are tested and reported	0.1	Before March 14
		0.3	On and after March 14
Shape	Weibull parameter in delay-to-reporting distribution	1.73 (1.60–1.86 95% CI)	Fit to data from Fig 1B
Scale	Weibull parameter in delay-to-reporting distribution	9.85 (9.30–10.46 95% CI)	Fit to data from Fig 1B
*R*_0b_	Basic reproductive number	2.95 (2.88–3.02 95% CI)	Fit to data from Fig 1C
*f*_2_	Fraction of normal contacts during physical distancing	0.22 (0.08– 0.36 95% CI)	Fit to data from Fig 1C
*ϕ*	Inverse dispersion from negative binomial (NB2) observation model	6.86 (3.39–12.37 95% CI)	Fit to data from Fig 1C

For some confirmed cases in BC, estimates of the date of symptom onset are available (Fig 1). We used the delays between symptom onset and cases being reported to parameterize the physical distancing model. In this case-specific data set there were only seven cases reported before February 29, and a decline in reported cases after April 2 (Fig 1). Therefore we used only the 535 cases in the case-specific data that were reported between these dates to parameterize the delay part of the model. For California (CA), New York (NY), and Florida (FL) we used reported case and testing data from The COVID Tracking Project, and assumed a BC-like delay between symptom onset and case reporting. For New Zealand (NZ) we used reported cases from the NZ Ministry of Health [18], and A. Lustig and M. Plank (pers. comm.) fit the delay distribution to NZ case-reporting data using our package [19], since these data are not publicly available (see Supplemental Methods in S1 Text).

For our main analysis with BC, we fit our model to data until April 11, 2020. When demonstrating the application of our model to other jurisdictions, we included data until May 6 or 7, 2020, the date we completed this portion of the analysis and before these jurisdictions had begun relaxing physical distancing measures. An outbreak at a poultry plant in BC, combined with an expansion of testing in mid April, meant that in order to extend the BC model to May 6 or 7, we would have had to introduce changes to the methodology that would not be straightforward and would not be possible in other jurisdictions. These include, for example, modelling the poultry workforce’s interventions and contacts and the links between the outbreak and general community transmission, in concert with differential testing. We therefore limited the data used for the BC model to April 11.

We motivated the structure of our model based on a survey conducted by the Angus Reid Institute to examine how physical distancing measures changed behaviour in Canada (March 20–23, 2020; n = 1664; [20]). Responses indicated that there was a subset of the population believing that the response to the COVID-19 epidemic was “overblown”, who were less willing than others to engage in distancing behaviours. This motivated treating the distancing and non-distancing compartments of our model separately and assuming that ∼80% of individuals were able and willing to physically distance. We used the timing of known government interventions to inform the timing of behavioural changes, and verified these dates against publicly available mobility data for each region [21].

### Epidemiological model

**Fig 2:**
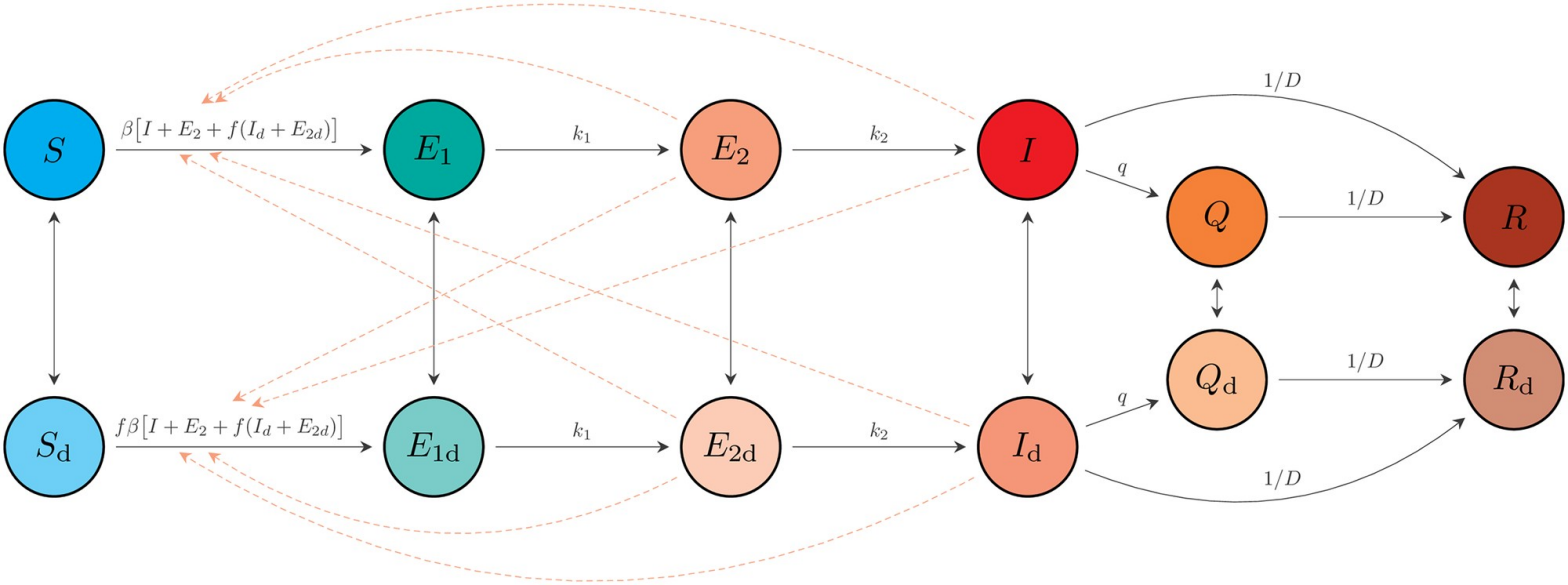
Schematic of the epidemiological model. Compartments are: susceptible to the virus (*S*); exposed (*E*_1_); exposed, pre-symptomatic, and infectious (*E*_2_); symptomatic and infectious (*I*); quarantined (*Q*); and recovered or deceased (*R*). Recovered individuals are assumed to be immune. The model includes analogous variables for individuals practising physical distancing: *S*_d_, *E*_1d_, *E*_2d_, *I*_d_, *Q*_d_, and *R*_d_. Solid arrows represent flow of individuals between compartments at rates indicated by the mathematical terms (see Supplement for full definitions). Dashed lines show which compartments contribute to new infections. An individual in some compartment *X* can begin distancing and move to the corresponding compartment *X*_d_ at rate *u*_d_. The reverse transition occurs at rate *u*_*r*_. The model quickly settles on a fraction *e* = *u*_*d*_/(*u*_*d*_ + *u*_*r*_) participating in distancing, and dynamics depend on this fraction, rather than on the rates *u*_*d*_ and *u*_*r*_.

We used a susceptible-exposed-infectious-recovered (SEIR) model. Our model allows for self-isolation and quarantine through a quarantine compartment and a reduced duration of infection (compared to the clinical course of disease). We modelled a fixed portion of the population that was able to participate in physical distancing; each of the SEIR compartments has an analogous compartment in the distancing group (Fig 2; Table 1; Supplemental Methods in S1 Text).

The physical distancing compartments contribute a reduced amount (a fraction *f*) to the force of infection, with *f* = 1 representing no physical distancing. We made *f* time-dependent to represent changes in physical distancing. We modelled the change in physical distancing by a simple linear function whereby physical distancing increases (*f*(*t*) decreases from 1 to a final value *f*_2_, which we estimated) over one week between March 15 (*t* = *t*_1_) and March 22 (*t* = *t*_2_) in BC, and as informed by policy and mobility data in the other jurisdictions (Table B in S1 Text; Eq. 3 in S1 Text). We fixed *f*(*t*) = *f*_2_ until the final day of observed data and changed *f*(*t*) for any future days to represent different scenarios regarding relaxation of physical distancing.

The number of people per day who become symptomatic is the number per day moving from the pre-symptomatic *E*_2_ and *E*_2*d*_ compartments to the symptomatic *I* and *I*_*d*_ compartments (Fig 2). Due to the delay between symptom onset and reporting, the model’s predicted number of reported cases, *μ*_*r*_, on day *r* is comprised of contributions from previous days weighted by a delay, which we estimate. We used a negative binomial model combined with information about testing changes over time to form a likelihood function, linking observed cases to the model (S1 Text).

The proportion of anticipated cases on day *r* that are tested and reported varies over time due to changes in the testing protocols, lab capacity, and other factors. In our BC data, the number of tests performed each day jumped dramatically on March 14; we modelled this with a sharp increase in *ψ*_*r*_ on that date. We fit a random walk to this function as a supplementary analysis. In other jurisdictions we fixed *ψ*_*r*_. We used a Weibull distribution for the delay function *w*(*s*), based on [22], and fit the shape and scale parameters using the case-specific data of reported cases and time of symptom onset (Fig 1), accounting for right truncation (S1 Text).

### Estimation

We used a Bayesian statistical model to condition our inference about *R*_0b_, *f*_2_, and expected case counts on the number of reported cases, where *R*_0b_ is the basic reproductive number. We related the expected number of cases to the observations through a negative binomial observation model with dispersion parameter *ϕ*. We placed weakly informative priors on *R*_0b_, *f*_2_, and *ϕ* (S1 Text). Outside of BC, we estimated the start and end times of the ramp-up of social distancing using priors informed by policy and transit data (S1 Text).

We fit our models with Stan 2.19.1 [30, 31] and R 3.6.2 [32]; Stan implements the No-U-Turn Hamiltonian Markov chain Monte Carlo algorithm [33] for Bayesian statistical inference. In our main model run, we sampled from eight chains with 2000 iterations each and discarded the first 1000 iterations of each chain as warm-up. We assessed chain convergence visually via trace plots (Figure G in S1 Text) and via ensuring that $\hat{R}\leq 1.01$ (the potential scale reduction factor) and that ESS > 200 (the effective sample size), as calculated by the rstan R package [31] (Tables C and D in S1 Text). We represent uncertainty via quantile-based credible intervals. We validated our approach using simulated data (S1 Text).

We developed two R packages for this analysis: ‘rightTruncation’ [19] and ‘covidseir’ [34]. ‘rightTruncation’ performs maximum likelihood estimates of delay distributions accounting for right truncation. We used this approach to estimate the time between symptom onset and reporting in BC and New Zealand. The ‘covidseir’ package facilitates the SEIR model fitting of case-count data in Stan.

## Results

**Fig 3:**
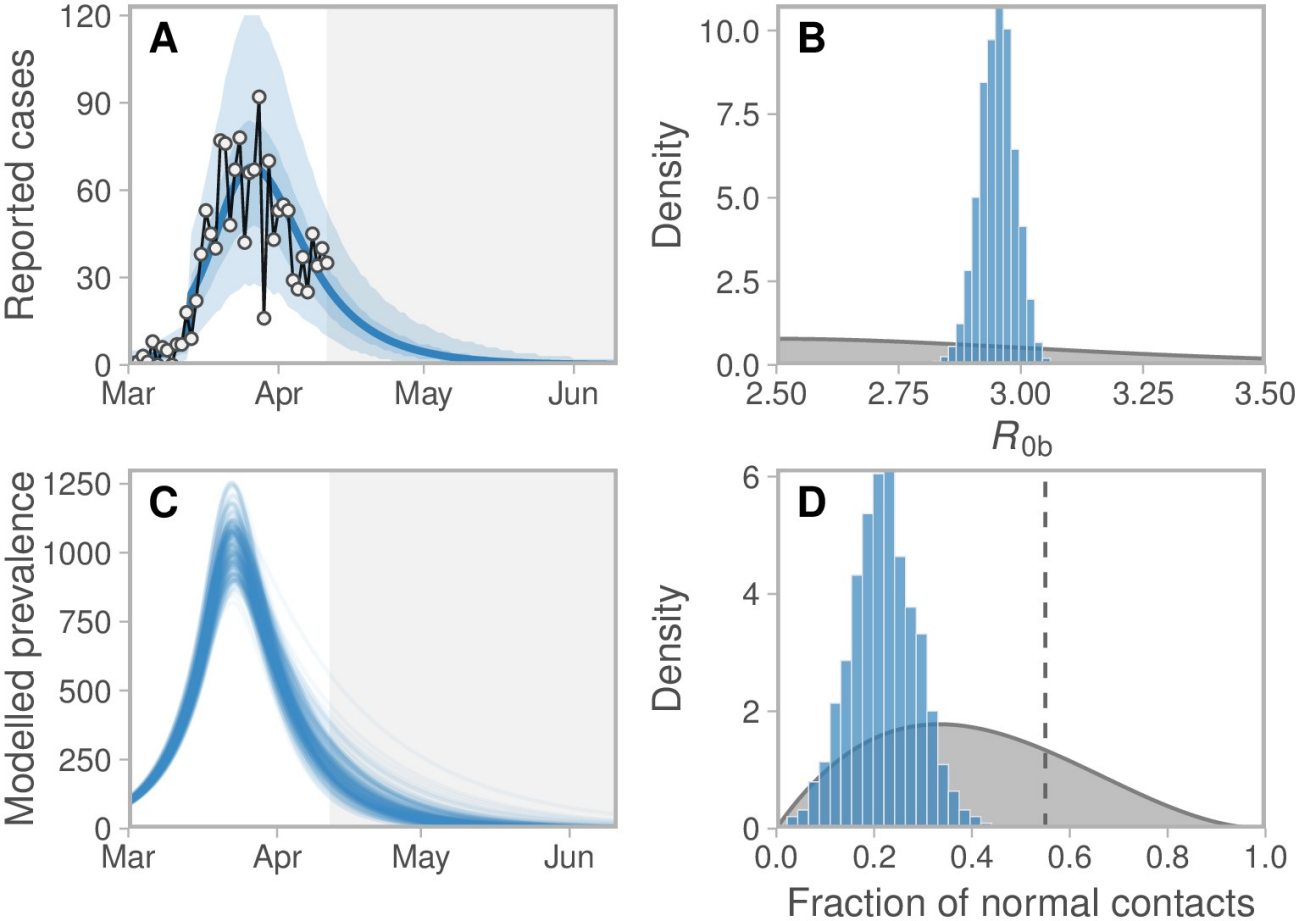
(A) Observed and estimated case counts, (C) estimated prevalence, and posterior estimates for (B) *R*_0b_ and (D) fraction of normal contacts (*f*_2_) among those distancing. These projections do not account for introduced cases from other jurisdictions and they assume that distancing measures remain in place. The fraction of normal contacts is the model’s portion of contacts that remain among those who are engaged in physical distancing. In panel A, the blue line represents the posterior mean and the shaded ribbons represent 50% and 90% credible intervals on new observations. Dots and black lines represent the reported data. Grey region indicates the projection. In panel C, lines represent example draws from the posterior. In panels B and D, priors are shown in grey and posteriors in blue. In panel D, the dashed vertical line denotes the threshold above which an exponential increase in prevalence is expected (see Figure J in S1 Text). **Note**: Model prevalence depends on assumptions about underestimation, incubation period, and the duration of infection, none of which we can estimate well from these data (Figure M in S1 Text). Much higher values of the prevalence are consistent with our data.

We found that, as of April 11, 2020, physical distancing had considerably reduced the contact rate in BC. We estimated that individuals practising physical distancing experienced approximately 0.22 (0.11–0.34 90% CI [credible interval]) of their normal contact rate, which was below the critical threshold (0.55; Fig 3; Figures E–H and Table C in S1 Text). The model described the count data well, with reported cases showing a peak in late March, approximately two weeks after the initiation of distancing measures (Fig 3A). The data were informative with respect to both main parameters with the posteriors distinctly different and more peaked than the priors (Fig 3B and 3D). We used a fixed value of *e*, the fraction engaged in distancing; this choice was motivated by the survey and behavioural data. If *e* were lower, the estimated strength of distancing would be higher to achieve the same case counts. We found this trade-off analytically using the basic reproduction number for the full model (Supplemental Methods and Figure A in S1 Text).

We found that with a shorter incubation period and duration of infectiousness, a lower reproduction number would fit the same overall growth rate, and conversely if the infectious duration and serial interval were longer, a higher reproduction number would be required but the fit to data would be similar (Figure K in S1 Text); this relationship is well known [35]. The conclusion that distancing measures reduced contact is robust to these alternatives (Figure K in S1 Text). The model depends on the fraction engaged in distancing, but not strongly on the rates *u*_*d*_ and *u*_*r*_; Figure L in S1 Text illustrates that we obtained the same results with these rates increased by a factor of 10. We also explored the robustness to the unknown underestimation fractions (Figure M in S1 Text), and a random walk pattern in the fraction of cases sampled (Figure N in S1 Text); again, the data are consistent with a range of underestimation fractions, but the conclusion about the contact fraction is robust.

**Fig 4:**
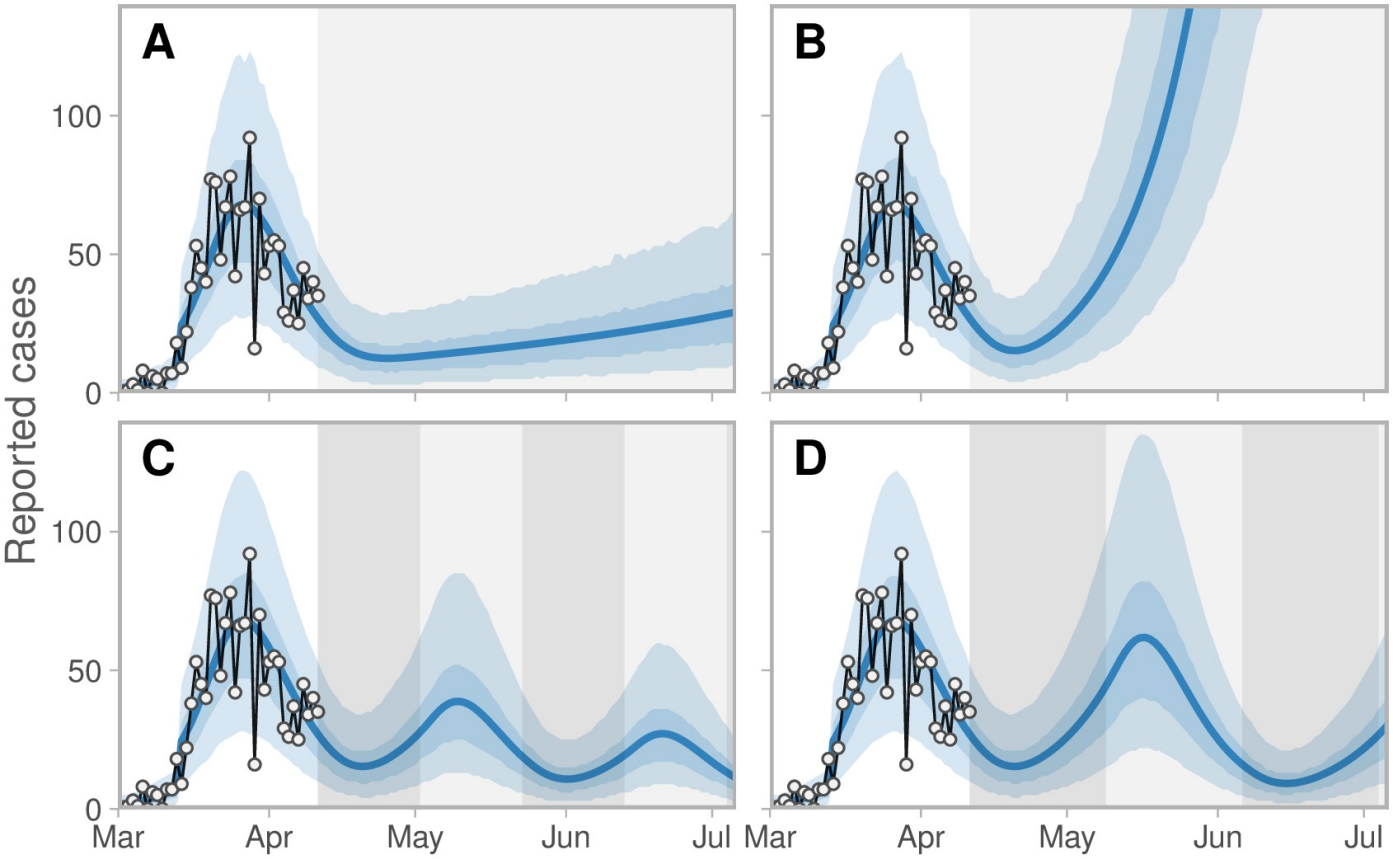
Scenarios of relaxing distancing measures. Distancing measures are relaxed to (A) 60% (A) and (B) 80% levels of normal contacts and exponential growth is observed at moderate and rapid rates. (C, D) Two scenarios of cycling between physical distancing levels. Here, the percentage of normal contacts alternates between 80% (dark-grey shading) and 22% (light-grey shading) at (C) 3-week and (D) 4-week intervals. Reducing contacts to 22% of normal is approximately the level estimated by our model (Fig 3). Note the lag between changes in physical distancing and reported case counts. Figure description is otherwise the same as for Fig 3A and 3C.

Our estimates suggest that, as of April 11, 2020, some relaxation of current distancing measures in BC would have been possible without bringing the growth rate above zero, but if measures were relaxed too much (in the absence of monitoring and re-starting measures), the prevalence and case counts would begin to increase exponentially (Fig 4A and 4B), reaching high levels by June 2020 if distancing were to cease entirely (Figure I in S1 Text). These are illustrative scenarios only; public health responses with renewed or revised measures would likely be put in place rapidly were such rises to be observed. The speed of growth depends on how close the system is to the epidemic threshold (Figure J in S1 Text). If strong enough measures are not maintained, the model predicts a range of possible epidemic curves (Figure I in S1 Text) consistent with simple and complex published models [11, 12, 14].

There has been interest in relaxing distancing measures and re-introducing them when a threshold has been reached, such as when intensive care capacity is nearly reached [36]. We did not explore a dynamic trigger, but did explore the behaviour when distancing measures are introduced and relaxed repeatedly (Fig 4C and 4D). If the relaxation period is such that the outbreak remains contained throughout (with an effective reproduction number less than one), then the prevalence would decline at alternating faster and slower rates. In a scenario of switching between the current mean estimate (22% of normal contacts) and 80% of normal contacts, reported cases rise, lagging the relaxation of distancing (Fig 4C and 4D). Illustrative simulations in which distancing alternates every three or four weeks allows an overall continued decline; however, the longer the period of relaxation, the more the prevalence is able to rise in between periods of distancing (Fig 4C and 4D). Control of delayed feedback systems is challenging [37], and ideally if a dynamic trigger such as reported cases or ICU admissions were to be used, monitoring would need to be as rapid as possible. Monitoring of distancing behaviour and population contact patterns would be important, in addition to monitoring cases.

**Fig 5:**
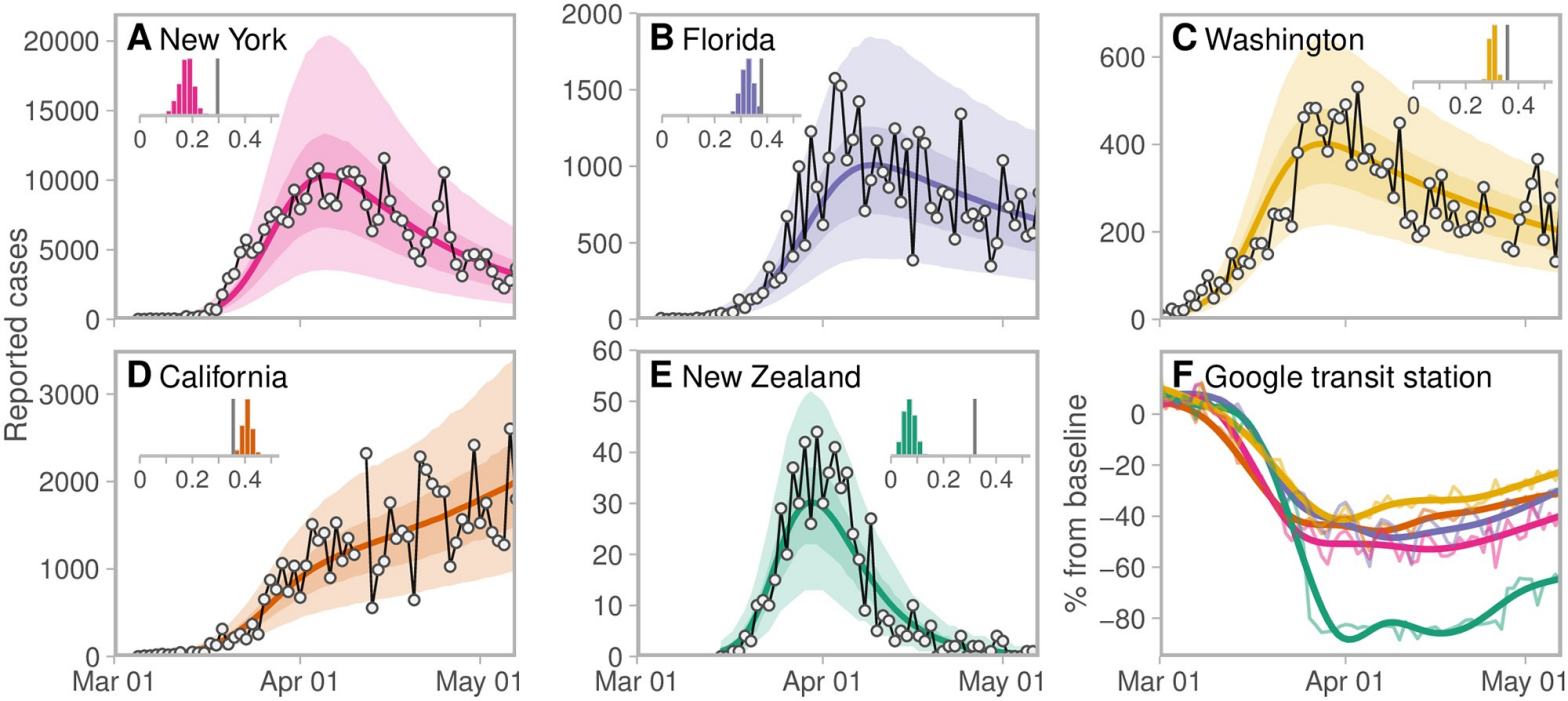
Observed and estimated case counts for (A) New York, (B) Florida, (C) Washington, (D) California, and (E) New Zealand. Solid curves represent the posterior means and shaded ribbons represent 50% and 90% credible intervals of estimated counts. Dots and black lines represent the reported data. Inset histograms show the posterior distributions of the fraction of normal contacts (*f*_2_), with the vertical lines denoting the threshold above which an exponential increase in prevalence is expected (as in Fig 3D). (F) Reduction in movement from Google mobility transit-station data [21] colour-coded for each jurisdiction. Thin lines are raw data; thick lines are smoothed values from a generalized additive model. See Table B and Supplemental Methods in S1 Text for details on the regional modelling parameters and initialization.

We estimated the fraction of normal contact rate in five additional jurisdictions as of May 7, 2020 (Fig 5), and give numerical results as the median (and 90% CI) of the “contact ratio”: the ratio of the fraction of normal contacts to the threshold (above which prevalence increases) for each jurisdiction. We found that while New York had a high peak in reported cases, overall control there was strong as of May 7 and there may have been room to relax distancing measures while remaining below the threshold above which cases would be expected to increase, since the contact ratio is 0.60 (0.43–0.74). We estimated contact ratios of 0.86 (0.76–0.96) for Florida and 0.84 (0.79–0.90) for Washington as of May 7. These are not far below 1.0, and so any re-opening or relaxation of distancing measures would be expected to result in rising case numbers. In contrast, while some areas in California experienced strong distancing and mobility data suggest movement on par with Florida, New York, and Washington (Fig 5F), overall case counts in California had not declined as of May 7, and our model estimated that contacts were exceeding the critical threshold on average (contact ratio of 1.15 [1.07–1.23]). In contrast, New Zealand had extremely effective control measures and we estimated that nearly all contacts were removed among those distancing as of May 6, 2020, with a contact ratio of 0.22 (0.11–0.34). This left considerable room for re-opening (in concert with careful border measures and continued contact tracing and case finding).

## Discussion

Our results suggest that physical distancing measures were effective in British Columbia; we estimated that individuals practising physical distancing in British Columbia to be experiencing approximately 0.22 (0.11–0.34 90% CI) of their normal contact rate as of April 11, 2020. This was below the threshold of 0.55 at which prevalence was expected to grow, which left some room to relax distancing measures. These results were supported by declines in hospitalizations and ICU admissions. We estimated that there was varying room to relax measures in other locations as of May 7, 2020. Strong control in New Zealand suggested considerable scope for restrictions to be relaxed. However, we found that there was relatively little room to relax measures in New York, Florida, and Washington. The overall picture in California, as of May 7, 2020, appeared to be that contacts were above the threshold that leads to increasing prevalence, and hence restrictions were not sufficient to curb spread of the disease. We note that in California, and all locations considered, it is likely that there was strong regional variation around our broad estimates. Our estimates for BC are consistent with local mobility data, and with contact patterns in other international locations. For example, survey data from the UK [39] suggested a 73% reduction in contacts, and a modelling study found that a 70–80% reduction in contacts is consistent with data in France [13].

Our estimate of the effect of distancing on contact patterns in BC is consistent with independent lines of evidence for the strength of distancing measures. Local rail (SkyTrain) station crowding data, provided by Metro Vancouver’s transportation authority TransLink, gives a proxy for reduction in public transport use. Overall daytime travel was reduced by 16% for the week of March 9, 64% for the week of March 16, and 73% for the week of March 23. Estimates on adhering to physical distancing are also available from a publicly available respondent-driven survey [20]. The survey found the rate of respondents stating that there was a serious threat of a coronavirus outbreak in Canada increased from 42% on March 5–6 to 88% on March 20–23. For individuals who stated there was a serious threat, 89% stated they were keeping personal distance compared to 66% for individuals who did not believe there was a serious threat. Mobile phone location data from BlueDot [39] suggested that the maximum and cumulative distance travelled from home fell by approximately 90%, the portion of mobile phone check-ins at home rose by over 10%, and the portion of devices for which every check-in was at home rose by 60%. These estimates are also consistent with Google mobility [21], Citymapper index [40], and Apple mobility data [41]. These are indirect reflections of the contact rate but are supportive of a dramatic change in contact patterns as reflected in our model. Furthermore, similar data are widely available for many jurisdictions. Our results provide a direct estimate of contact rates in conjunction with mobility data.

Our results suggest that some relaxation of distancing measures may have been possible in BC and New York, considerably so in New Zealand, but that relaxation would have been risky in Washington and Florida. In BC, we simulated fixed and dynamic measures—less stringent than the measures in place at present—which would continue to maintain low case numbers. This is feasible either through continual strong distancing, or via well-monitored dynamic on/off measures. We have illustrated the model’s high case volumes and long time frames that would result from cessation of distancing and the absence of continued strong public health and behavioural intervention in BC; the dynamics would look similar elsewhere. In all jurisdictions, we found that immunity has not built up in the model; our estimates of the decline are not due to a natural peak in an epidemic curve, but are the direct effect of changes in contact patterns. Seasonal transmission could even amplify a peak in the winter season, if control and monitoring were ceased then [12].

The delays between exposure and reporting present obvious challenges for monitoring the success of control measures using reported case-count data. We suggest that two different kinds of monitoring will be important if distancing measures are to be relaxed: (1) monitoring cases through testing, contact tracing, and other case finding, and (2) monitoring contact patterns and distancing behaviour in the population, in a “distancing surveillance” effort. This latter form of monitoring, derived from mobile phones, surveys and apps, could be available rapidly, whereas the incubation period places an unavoidable delay between control measures and detecting their impact in reported cases—even if testing of symptomatic cases were widespread and reporting were immediate. There is considerable interest in real-time monitoring of mobile phone movements, population surveys on the uptake of physical distancing, and other behavioural data. While these are potentially promising avenues, the outcome of interest is incident infections. Locations of mobile phones, traffic patterns, and survey information are proxies for this outcome at best. The work we have presented here could help to calibrate distancing surveillance measurements, to understand how they relate to changes in contact rates for modelling efforts.

Our modelling framework has a number of important limitations. We do not model age and contact structure explicitly, except to distinguish between two populations: those participating in distancing or not. This has the advantage that we do not require data on age-specific contact patterns, responses to distancing measures, or infectiousness; these data are not available at this time. It also limits our ability to provide guidance on where and how contact reduction measures could be implemented. It is a simplification of behaviour in many ways; true distancing responses are a continuum, and the measures in place (e.g., no mass gatherings or dine-in services) also mean that the whole population is experiencing some changes in contact patterns. Our model is deterministic, and so does not capture the possibility of extinction; in addition, we have not simulated introductions of COVID-19 from other jurisdictions. We have not accounted for geographic structure; differences in distancing behaviour, health care practices, and demographics in different jurisdictions could impact the results. We have also not modelled either conventional or automated contact tracing [42, 43]; in our model these would decrease the duration of the infectious period and change the transitions for some exposed individuals.

There are also limitations in our data. We have used an observation model to link reported cases to the modelled prevalence, and we included variation in the portion of cases detected over time. Modelling and forecasting based on reported cases faces challenges when testing is driven by clinical needs, testing capacities, and other constraints (and in particular is not designed to test population samples). Cases in long-term care facilities (LTCF) represent a substantial fraction of the cases, and particularly the deaths, in BC. Along with the low number of deaths in total, this is one rationale for not modelling deaths explicitly. We included LTCF cases but also modelled a wide range of under-reporting to account for potential biases. If many cases in an LTCF cluster were all reported on the same day (or within a short time frame) this could increase the noise in reported case counts. We have modelled case counts as over-dispersed compared to a Poisson distribution to account for such variation; we have developed the R package to model a range of data types individually or in combination (e.g., reported cases, hospitalizations, ICU admissions), which could help to overcome limitations of particular data.

The testing criteria include a number of categories that have changed over time. Testing volume increased sharply in mid March in BC and most jurisdictions have changed testing volumes. The base population being tested has also changed. In BC, testing first focused primarily on those hospitalized or likely to be hospitalized, health care workers, residents of long-term care facilities, and other cluster investigations (mid-March to April 9). Testing was then expanded to include residents of remote, isolated, or Indigenous communities, people who are homeless or have unstable housing, and by physicians’ clinical judgement. There is likely some inconsistency in the application of these guidelines across hospitals and facilities, and base populations differ in across jurisdictions as well. As a consequence, the base population being tested changes with time and contributes a changing portion of the force of infection. Underestimation is therefore complex and is comprised of varying under-ascertainment and under-reporting.

There remain important unknowns about COVID-19 that give rise to additional limitations for modelling efforts; immunity and asymptomatic transmission are two of these. We have included pre-symptomatic transmission but we have not explicitly modelled asymptomatic individuals, who may have few or no symptoms but nonetheless be transmitting, and who may or may not be building lasting immunity. Recent work suggests that both pre- and asymptomatic individuals may be contributing considerably to transmission [27, 28, 44]. We have indirectly approached this uncertainty, exploring variable underestimation fractions and duration of the incubation and infectious periods. A wide range for underestimation and duration is consistent with the reported case data. Our conclusion about the impact of distancing measures appears to be robust to these uncertainties, although the basic reproductive number and the model prevalence vary according to assumptions about underestimation and duration. Model predictions for the peak timing and size of prevalence without strong public health interventions will depend strongly on the dynamics of immunity, including the numbers of asymptomatic individuals and their immunity [12].

Our model suggests that distancing measures were working well in British Columbia as of April, 2020, that some relaxation of these measures may have been possible, but that this must be done carefully. More broadly, we estimated a range of effectiveness of physical distancing in other locations (from very strong in New Zealand to weak in California as of May 7). Given the likely low levels of immunity, long-term public health measures will be necessary to control COVID-19. If data were available describing contact patterns and transmissibility by age, along with data describing the impact of specific activities on these contact patterns, then models could be effective tools to determine how to safely relax distancing measures—for example, restarting specific activities such as particular workplaces or schools. Without knowledge of prevalence and transmissibility in children, of the extent of asymptomatic infection, and of the contact patterns that would result from restarting specific activities, models aiming to simulate these activities will have large uncertainties. We therefore suggest that there is an urgent need for longitudinal measurements of population-level prevalence and immunity via viral testing and serological studies, even where prevalence is low. Furthermore, if distancing measures are to be relaxed, it will be crucial to have strong surveillance through widespread testing, contact tracing, and isolation of new cases, as well as strong compliance with potentially shifting public health policy and messaging.

## Supporting information

S1 Text Model specification, validation and analysis, likelihood parameter estimation, supplemental results, sensitivity analysis. (PDF)
