# Supplementary material for: Quantifying the impact of COVID-19 control measures using a Bayesian model of physical distancing
Source: PLoS Comput Biol. 2020 Dec 3;16(12):e1008274. doi: 10.1371/journal.pcbi.1008274 (PMC7738161; doi:10.1371/journal.pcbi.1008274)
Supplement: S1 Text — (PDF) [file pcbi.1008274.s001.pdf]

S1 Supporting Information: Quantifying the impact of COVID-19 control measures using a Bayesian model of physical distancing

Supplemental Methods

Full model definition

We developed an epidemiological model to describe the numbers of individuals who are: susceptible (S); exposed to the virus (E_1); exposed, pre-symptomatic, and infectious (E_2); symptomatic and infectious (I); quarantined (Q); and recovered or deceased (R) over time (Fig 2). Recovered individuals are assumed to be immune. The model includes analogous variables for individuals practising physical distancing, represented by subscript d, i.e. S_d, $E_{1\mathrm{d}}$, $E_{2\mathrm{d}}$, I_d, Q_d, and R_d. Physical distancing reduces the frequency of contact between individuals (and hence between compartments; Fig 2).

The non-physical-distancing differential equations are:

$$
\begin{aligned}
\frac{\mathrm{d}S}{\mathrm{d}t} &= -\beta\left[I + E_2 + f(I_\mathrm{d} + E_{2\mathrm{d}})\right]\frac{S}{N} - u_\mathrm{d}S + u_r S_\mathrm{d} \\
\frac{\mathrm{d}E_1}{\mathrm{d}t} &= \beta\left[I + E_2 + f(I_d + E_{2\mathrm{d}})\right]\frac{S}{N} - k_1E_1 - u_\mathrm{d}E_1 + u_rE_{1\mathrm{d}} \\
\frac{\mathrm{d}E_2}{\mathrm{d}t} &= k_1E_1 - k_2E_2 - u_\mathrm{d}E_2 + u_rE_{2\mathrm{d}} \\
\frac{\mathrm{d}I}{\mathrm{d}t} &= k_2E_2 - qI - \frac{I}{D} - u_\mathrm{d}I + u_rI_\mathrm{d} \\
\frac{\mathrm{d}Q}{\mathrm{d}t} &= qI - \frac{Q}{D} - u_\mathrm{d}Q + u_rQ_\mathrm{d} \\
\frac{\mathrm{d}R}{\mathrm{d}t} &= \frac{I}{D} + \frac{Q}{D} - u_\mathrm{d}R + u_rR_\mathrm{d},
\end{aligned}
\tag{1}
$$

where β is the transmission parameter, D is the mean duration of the infectious period, f represents physical distancing, u_d and u_r are the rates of movement to and from the physical distancing compartments, k_1 is the rate of movement from the E_1 to E_2 compartment, k_2 is the rate of movement from the E_2 to I compartment, and q is the quarantine rate for movement from the I to Q compartment.

In the model without interventions (neither distancing nor quarantine), the basic reproductive number $R_{0\mathrm{b}}$ is $\beta(D+1/k_2)$, namely the transmission rate times the mean duration in the infectious compartment.

There are six analogous equations for the physical distancing compartments (denoted by subscript d). The physically distancing compartment S_d has a contact rate with infectious individuals that is a fraction f of that for the S compartment. This factor appears twice: distancing individuals contribute less to the force of infection and also are less likely to encounter others than

non-distancing individuals, giving

$$
\begin{aligned}
\frac{\mathrm{d}S_\mathrm{d}}{\mathrm{d}t} &= -f\beta\left[I + E_2 + f(I_\mathrm{d} + E_{2\mathrm{d}})\right]\frac{S_\mathrm{d}}{N} + u_\mathrm{d}S - u_r S_\mathrm{d}\\
\frac{\mathrm{d}E_{1\mathrm{d}}}{\mathrm{d}t} &= \ f\beta\left[I + E_2 + f(I_d + E_{2\mathrm{d}})\right]\frac{S_\mathrm{d}}{N} - k_1E_{1\mathrm{d}} + u_\mathrm{d}E_1 - u_rE_{1\mathrm{d}}\\
\frac{\mathrm{d}E_{2\mathrm{d}}}{\mathrm{d}t} &= k_1E_{1\mathrm{d}} - k_2E_{2\mathrm{d}} + u_\mathrm{d}E_2 - u_rE_{2\mathrm{d}}\\
\frac{\mathrm{d}I_\mathrm{d}}{\mathrm{d}t} &= k_2E_{2\mathrm{d}} - qI_\mathrm{d} - \frac{I_\mathrm{d}}{D} + u_\mathrm{d}I - u_rI_\mathrm{d}\\
\frac{\mathrm{d}Q_\mathrm{d}}{\mathrm{d}t} &= qI_\mathrm{d} - \frac{Q_\mathrm{d}}{D} + u_\mathrm{d}Q - u_rQ_\mathrm{d}\\
\frac{\mathrm{d}R_\mathrm{d}}{\mathrm{d}t} &= \frac{I_\mathrm{d}}{D} + \frac{Q_\mathrm{d}}{D} + u_\mathrm{d}R - u_rR_\mathrm{d}.
\end{aligned}
\tag{2}
$$

The distancing strength $(1 - f)$ changes with time as distancing measures ramp up:

$$
f(t) = \begin{cases} 1, & t < t_1,\\ f_2 + \dfrac{t_2 - t}{t_2 - t_1}(1 - f_2), & t_1 \le t < t_2,\\ f_2, & t \ge t_2. \end{cases}
\tag{3}
$$

We relate the observed cases to the model prevalence with an observation model in which the observed cases are overdispersed and are comprised of contributions from those who became symptomatic over the previous week. The expected number of observed cases on day r is

$$
\mu_r = \psi_r \int_0^{45} k_2\left[E_2(r - s) + E_{2\mathrm{d}}(r - s)\right] w(s)\mathrm{d}s,
\tag{4}
$$

where $w(s)$ is the density function for delay s and 45 is the maximum delay that needs to be considered.Also, ψ_r is the portion tested, which can change over time.

The number of reported cases each day is denoted C_r, where r represents discrete days (whereas t is continuous time in the model). We model dispersion using the NB2 parameterization of the probability mass function [1]:

$$
\mathrm{NB2}(C_r \mid \mu_r, \phi) = \binom{C_r + \phi - 1}{C_r}\left(\frac{\mu_r}{\mu_r + \phi}\right)^{C_r}\left(\frac{\phi}{\mu_r + \phi}\right)^{\phi}, \text{ for } r = 1, \ldots, 42,
\tag{5}
$$

in which the variance scales quadratically with the mean according to the (inverse) dispersion parameter, ϕ: $\mathrm{Var}[\{C_r\}] = \mu + \mu^2/\phi$.

The model is fit to the data in a Bayesian context to estimate parameters $R_{0\mathrm{b}}, f_2$, and ϕ, with f_2 being of particular interest as the fraction of normal contacts for those individuals who are physical distancing. The joint posterior distribution given the case counts $\{C_r\}$ is

$$
\mathrm{P}\left(R_{0\mathrm{b}}, f_2, \phi | \{C_r\}\right) \propto \mathrm{P}\left(\{C_r\} | R_{0\mathrm{b}}, f_2, \phi\right)\mathrm{P}(R_{0\mathrm{b}})\mathrm{P}(f_2)\mathrm{P}(\phi),
\tag{6}
$$

where the term $\mathrm{P}(\{C_r\}|R_{0\mathrm{b}}, f_2, \phi)$ refers to the negative binomial data likelihood with (inverse) dispersion parameter ϕ, and the terms $\mathrm{P}(R_{0\mathrm{b}})$, $\mathrm{P}(f_2)$, and $\mathrm{P}(\phi)$ refer to prior distributions.

We placed weakly informative priors on R_{0b}, f_2, and ϕ as follows. We chose a prior on R_{0b} to encompass values commonly published in the literature for SARS-CoV-2 [2, 3]: $\text{Lognormal}(\log(2.6), 0.2)$. We chose a prior for f_2 that resulted in a mean of 0.4 and a standard deviation of 0.2, $f_2 \sim \text{Beta}(2, 3)$, to represent a moderately strong reduction in contact fraction while still being broad enough to encompass a wide range of values. We chose a prior on ϕ to constrain the model to avoid substantial prior mass on a large amount of over-dispersion (small values of ϕ): $1/\sqrt{\phi} \sim \text{Normal}(0, 1)$ [4].

Initialization

Specifying initial conditions for the epidemiological model involves setting a total number of infected people I_0 at an initial point in time (February 1, 2020 for BC), which are divided among the exposed and infected compartments. We define $e = u_\text{d}/(u_\text{d} + u_r)$ to be the fraction practising physical distancing ($e = 0.83$ in the base model). We derived this fraction distancing from the Angus Reid Institute survey [5]: 89% of the 88% who believed that COVID-19 was a serious threat were practicing distancing. There were three distancing questions: (1) keeping extra distance (89% among those who took COVID-19 to be a serious threat vs. 66% among those who did not); (2) not shaking hands or hugging (87% vs. 66%); and (3) avoiding public spaces (84% vs. 60%). If we take a weighted average of these numbers we get 0.84. If we also assume that those who do not take COVID-19 to be a serious threat perform distancing somewhat less diligently than those who do then we get our 0.83, which also is a number that arises from using round numbers for $u_r = 0.1$ and $u_d = 0.02$: $u_r/(u_r + u_d)$.

To further avoid sharp initial transient behaviour we also initially distribute all individuals among the distancing and non-distancing components, to give reasonable conditions for March 1 (Table A). The model begins on February 1 with 8 cases, reflecting a 10–30% chance of detection [6] and very low reported cases at that time. We do not model introductions during February, and instead we compensate for this with an elevated initial number of infectious cases.

Table A: Initial values

Non-distancing			Distancing		
Variable	Initial definition	Initial value	Variable	Initial definition	Initial value
S	$(1 - e)(N - I_0)$	849999	S_d	$e(N - I_0)$	4249993
E_1	$0.4(1 - e)I_0$	0.53	E_1d	$0.4eI_0$	2.67
E_2	$0.1(1 - e)I_0$	0.13	E_2d	$0.1eI_0$	0.67
I	$0.5(1 - e)I_0$	0.67	I_d	$0.5eI_0$	3.33
Q	0	0.00	Q_d	0	0
R	0	0.00	R_d	0	0

Determining the threshold contact rate

To determine the threshold fraction of normal contacts that would lead to expected increases in prevalence, we applied the following approach (Figure J). (1) For a sequence of fractions of

normal contacts (0.2, 0.4, 0.6, 0.8, 1.0), project the model posterior for 60 days into the future. (2) Fit a linear regression to determine the slope of log(prevalence) vs. time (day) for the last 30 days of the projection period for each of the fractions of normal contacts. (3) Fit a linear regression to the slopes from the previous step against the fraction of normal contacts. (4) Use this fitted regression line to determine what fractional normal contacts would result in an expected change in log prevalence of zero over time based on where the regression line crosses 0 on the y-axis (Figure J).

Model validation

To test the model's ability to recover the parameters and to test our implementation of the Stan model, we repeatedly fit the model to data simulated from separate code in R. We inspected the resulting posterior distributions for bias and coverage of the known true values and visually inspected the resulting time series (e.g. Figure C and Figure D). Furthermore, we conducted posterior predictive checks [7] to assess whether the observed data were consistent with data generated by the model. Code to reproduce our analysis is available at `https://github.com/carolinecolijn/distancing-impact-covid19`.

Regional modelling parameters and initialization

Table B: Regional modelling initialization, data properties, and priors.

	BC	NY	FL	WA	CA	NZ
First day of data	Mar 1	Mar 4	Mar 5	Mar 1	Mar 5	Mar 15
Last day of data	Apr 11	May 7	May 7	May 7	May 7	May 6
I_0: incidents 30 days before day 1	8	0.5	1	1	1	0.001
f_2 prior: Beta(mean, SD)	0.4, 0.2	0.4, 0.2	0.4, 0.2	0.4, 0.2	0.4, 0.2	0.3, 0.2
R_{0b} prior: Lognormal(logmean, SD)	2.6, 0.2	2.6, 0.2	2.6, 0.2	2.6, 0.2	2.6, 0.2	2.6, 0.2
$1/\sqrt{\phi}$ prior: Normal(mean, SD)	0, 1	0, 1	0, 1	0, 1	0, 1	0, 1
f_2 ramp starting day prior mean	Mar 15	Mar 13	Mar 16	Mar 11	Mar 12	Mar 18
f_2 ramp ending day prior mean	Mar 22	Mar 28	Mar 28	Mar 23	Mar 24	Mar 26
Proportion distancing: $u_r/(u_r + u_d)$	0.83	0.83	0.83	0.83	0.83	0.95
Delay shape	1.73	1.73	1.73	1.73	1.73	1.53
Delay scale	9.85	9.85	9.85	9.85	9.85	7.83
Sampling fraction	0.1/0.3	0.25	0.25	0.25	0.25	0.40
N: total population (millions)	5.1	19.45	21.48	7.6	39.51	4.95

The physical distancing "ramp" (linear change in f_2) starting and ending dates given in Table B refer to lognormal mean prior dates; the standard deviation was 0.1 for each region. Start and end priors were derived from policy events combined with inspection of the Google transit station data[8], except in BC where we fixed the start and end dates. In all regions but BC and New Zealand we did not have data for the distribution of the delay time between symptom onset and case reporting, so we used BC parameters. Our fits for the start and end of the distancing ramp

therefore account for the use of BC delay shape and scale parameters, which may not match testing in these regions. They also must account for fitting a linear physical distancing ramp to a process that may not be linear for all regions (e.g., Fig 5F).

We obtained US data from The COVID Tracking Project [9] on May 7, 2020, and data from NZ were obtained from the New Zealand Ministry of Health [10]. We do not have data to inform the initial introduction of COVID-19 to jurisdictions, and the ODE modelling framework is not well-suited to capture individual introductions and their secondary cases, so the initialization of the model is necessarily approximate. We set an initial number of prevalent cases based on the models' fit for the first days of reported cases. We also do not have data on the portion of the population engaged in distancing; this was set to BC values except for NZ where we assumed that the strict physical distancing policies would result in a very high proportion of individual distancing. Increasing the fraction to 0.95 improved the model fit, allowing cases to rise and fall at the fast rate observed in the data. While the values of f_2 that we estimate for the jurisdictions likely depend on the fraction involved in distancing, the distance between the estimate and the threshold is robust to this (Figure A illustrates the trade-off; changing the fraction distancing would change f_2 *and* the threshold).

In BC we had information about changes in testing over time, and we did not have this in other jurisdictions, so the sampling fractions were fixed over time. This affects the modelled prevalence but does not impact the conclusion about the contact fraction (Figure M). We used the same priors for R_0 and ϕ as for BC, and other initialization as specified in Table A.

Computing R_0 analytically

We compute the basic reproduction number R_0 for the full model, as a function of the model parameters, using the Next-Generation method [11, 12]. We set f to be constant, dropping the subscript 2, and set $u_{\mathrm{d}} = 1$, $e := u_{\mathrm{d}}/(u_r + u_{\mathrm{d}})$, and $u_r = (1-e)/e$. The disease-free equilibrium is $\epsilon_0 = ((1-e)N, 0, 0, 0, 0, 0, eN, 0, 0, 0, 0, 0)$. We identify E_1, E_2, I, Q, $E_{1\mathrm{d}}$, $E_{2\mathrm{d}}$, I_{d}, Q_{d} as the infection compartments, and denote the corresponding system by $\mathscr{X}$.

We decompose $\mathscr{X} = \mathscr{F} - \mathscr{V}$, where

$$\mathscr{F} = \begin{bmatrix} \beta(I + E_2 + f(I_{\mathrm{d}} + E_{2\mathrm{d}}))\frac{S}{N} \\ 0 \\ 0 \\ 0 \\ f\beta(I + E_2 + f(I_{\mathrm{d}} + E_{2\mathrm{d}}))\frac{S_{\mathrm{d}}}{N} \\ 0 \\ 0 \\ 0 \end{bmatrix}, \quad \mathscr{V} = \begin{bmatrix} k_1E_1 + E_1 - \frac{(1-e)}{e}E_{1\mathrm{d}} \\ -k_1E_1 + k_2E_2 + E_2 - \frac{(1-e)}{e}E_{2\mathrm{d}} \\ -k_2E_2 + qI + \frac{I}{D} + I - \frac{(1-e)}{e}I_{\mathrm{d}} \\ -qI + \frac{Q}{D} + Q - \frac{(1-e)}{e}Q_{\mathrm{d}} \\ k_1E_{1d} - E_1 + \frac{(1-e)}{e}E_{1\mathrm{d}} \\ -k_1E_{1d} + k_2E_{2\mathrm{d}} - E_2 + \frac{(1-e)}{e}E_{2\mathrm{d}} \\ -k_2E_{2\mathrm{d}} + qI_d + \frac{I_d}{D} - I + \frac{(1-e)}{e}I_{\mathrm{d}} \\ -qI_{\mathrm{d}} + \frac{Q_{\mathrm{d}}}{D} - Q + \frac{(1-e)}{e}Q_{\mathrm{d}} \end{bmatrix}.$$

Linearization around ϵ_0 gives the following Jacobian matrices for $\mathscr{F}$ and $\mathscr{V}$ respectively,

$$F = \begin{bmatrix} 0 & \beta(1-e) & \beta(1-e) & 0 & 0 & f\beta(1-e) & f\beta(1-e) & 0 \\ 0 & 0 & 0 & 0 & 0 & 0 & 0 & 0 \\ 0 & 0 & 0 & 0 & 0 & 0 & 0 & 0 \\ 0 & 0 & 0 & 0 & 0 & 0 & 0 & 0 \\ 0 & f\beta e & f\beta e & 0 & 0 & f^2\beta e & f^2\beta e & 0 \\ 0 & 0 & 0 & 0 & 0 & 0 & 0 & 0 \\ 0 & 0 & 0 & 0 & 0 & 0 & 0 & 0 \\ 0 & 0 & 0 & 0 & 0 & 0 & 0 & 0 \end{bmatrix},$$

$$V = \begin{bmatrix} k_1+1 & 0 & 0 & 0 & -\frac{(1-e)}{e} & 0 & 0 & 0 \\ -k_1 & k_2+1 & 0 & 0 & 0 & -\frac{(1-e)}{e} & 0 & 0 \\ 0 & -k_2 & q+\frac{1}{D}+1 & 0 & 0 & 0 & -\frac{(1-e)}{e} & 0 \\ 0 & 0 & -q & \frac{1}{D}+1 & 0 & 0 & 0 & -\frac{(1-e)}{e} \\ -1 & & 0 & 0 & k_1+\frac{(1-e)}{e} & 0 & 0 & 0 \\ 0 & -1 & 0 & 0 & -k_1 & k_2+\frac{(1-e)}{e} & 0 & 0 \\ 0 & 0 & -1 & 0 & 0 & -k_2 & q+\frac{1}{D}+\frac{(1-e)}{e} & 0 \\ 0 & 0 & 0 & -1 & 0 & 0 & -q & \frac{1}{D}+\frac{(1-e)}{e} \end{bmatrix}.$$

We use SageMath [13] via CoCalc [14] to compute the spectral radius of FV^{-1} and simplify its expression. We find that

$$\begin{aligned} R_0 = {} & \beta\frac{e^4(1-e)(1-f)^2k_1k_2}{(e(\frac{1}{D}+q)+1)(ek_1+1)(ek_2+1)} + \beta\frac{(ef+1-e)^2}{\frac{1}{D}+q} + \\ & \beta\frac{ek_1(ef+1-e)^2}{k_2(ek_1+1)(ek_2+1)} + \beta\frac{e(ef+1-e)^2}{(ek_1+1)(ek_2+1)} + \\ & \beta\frac{(ef+1-e)^2}{k_2(ek_1+1)(ek_2+1)} + \beta\frac{e^2k_1(ef^2+1-e)}{(ek_1+1)(ek_2+1)}. \end{aligned} \tag{7}$$

Figure A shows an example of the relation between R_0 and the model parameters. The fixed parameters are taken from Table 1, with β computed as $R_{0\mathrm{b}}/(D+1/k_2)$.

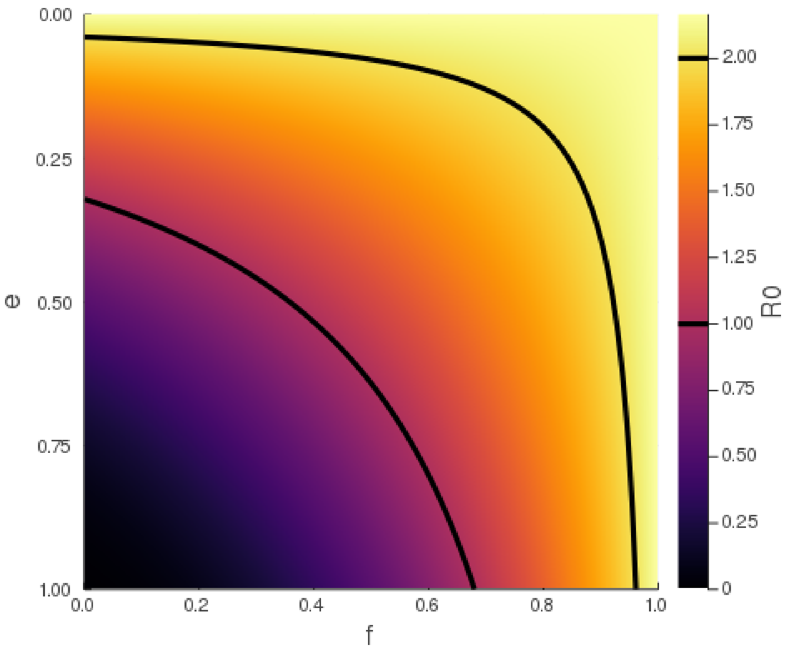

Figure A: R_0 as a function of e and f, with fixed parameters $\beta = 0.433$, $D = 5$, $q = 0.05$, $k_1 = 0.2$, and $k_2 = 1$. The black contours represent $R_0 = 1$ and $R_0 = 2$, respectively.

Likelihood estimation of parameters for the delay distribution

We derive a likelihood function to estimate the shape and scale parameters of the Weibull delay distribution using the case-specific data that are available for some individual cases. These data are right-truncated (Fig 1), in that we are not aware of cases that will be reported in the future, yet their symptoms have already occurred in the past. This is dealt with by the likelihood function. The resulting maximum likelihood estimates are then used in the observation model to relate reported cases to model prevalence. We developed an R package 'rightTruncation' to make implementation of these likelihood methods available for wider use [15].

The data on individual cases in BC (Fig 1) give counts h_{nr} of the number of individuals whose case was reported (test was positive) at the end of day r and whose symptoms are estimated to have started on day n. If day N is the final day of these data, then the full data set is $\{h_{nr}\}_{n=0,1,2,\ldots,N; n \leq r \leq N}$, where, by definition, $h_{nr} = 0$ for $r < n$ because a case cannot be reported before the start of symptoms for these data. The counts are right-truncated on day N, since there are individuals whose symptoms started on day n who will be reported in the future (after N), but we do not yet know when. The cases are considered to be reported at the end of day r because there are values of $h_{nn} > 0$.

We assume a Weibull distribution for the delay time between symptom onset and reporting of a case. A Weibull distribution was motivated by findings [16] that it was a better fit than the gamma or lognormal distributions to early data from Wuhan, China, on incubation periods of COVID-19, though note that we are fitting delay to reporting not the incubation period. Furthermore the Weibull parameters have a direct interpretation in terms of a failure model; we find that the shape parameter is > 1, indicating that the probability of reporting increases with time, consistent with, for example, worsening symptoms. Then the probability of a case whose symptoms start on day n being reported on day $r \geq n$ is

$$p'_{nr} = \int_{r}^{r+1} w(\tau' - n; \lambda, k) \mathrm{d}\tau' \tag{8}$$

$$= \int_{r-n}^{r-n+1} w(\tau; \lambda, k) \mathrm{d}\tau, \tag{9}$$

where the probability density function for the Weibull distribution with shape $k > 0$ and scale $\lambda > 0$ is

$$w(\tau; \lambda, k) = \begin{cases} \dfrac{k}{\lambda} \left(\dfrac{\tau}{\lambda} \right)^{k-1} \mathrm{e}^{-(\tau/\lambda)^k}, & \tau \geq 0, \\ \\ 0, & \tau < 0. \end{cases} \tag{10}$$

The integral in (9) is needed because the Weibull is a continuous function of time (and time is continuous in the differential equation model). We define the cumulative distribution function as

$$W(x; \lambda, k) = \mathrm{P}(X \leq x; \lambda, k) = \int_{0}^{x} w(\tau; \lambda, k) \mathrm{d}\tau. \tag{11}$$

Then

$$p'_{nr} = W(r - n + 1) - W(r - n), \tag{12}$$

where we have dropped the explicit λ and k in $W(\cdot)$ for clarity.

Equation (9) holds for $r > N$, but the data are only available up to $r = N$. Consider that today is day $N = 5$, then we have a matrix of p'_{nr} values:

$$(p'_{nr}) = \begin{pmatrix} p'_{00} & p'_{01} & p'_{02} & p'_{03} & p'_{04} & p'_{05} & | & p'_{06} & p'_{07} & \cdots \\ 0 & p'_{11} & p'_{12} & p'_{13} & p'_{14} & p'_{15} & | & p'_{16} & p'_{17} & \cdots \\ 0 & 0 & p'_{22} & p'_{23} & p'_{24} & p'_{25} & | & p'_{26} & p'_{27} & \cdots \\ 0 & 0 & 0 & p'_{33} & p'_{34} & p'_{35} & | & p'_{36} & p'_{37} & \cdots \\ 0 & 0 & 0 & 0 & p'_{44} & p'_{45} & | & p'_{46} & p'_{47} & \cdots \\ 0 & 0 & 0 & 0 & 0 & p'_{55} & | & p'_{56} & p'_{57} & \cdots \end{pmatrix}. \tag{13}$$

The probabilities in each row sum to 1 (since every case will eventually become reported, else it would not become a 'case'). The cases to the right of the vertical dashed line are the ones that we do not yet have data on; for example, people whose symptoms started on day 1 and whose case will be reported tomorrow (day 6, i.e. p'_{16}).

We need to calculate p_{nr}, the probability that a case that is reported on day r exhibited the onset of symptoms on day n. This requires taking the values in the above matrix that are to the left of the dashed line, and normalising each column such that it sums to 1, i.e.

$$p_{nr} = \frac{p'_{nr}}{\sum_{i=0}^{r} p'_{ir}}, \quad n, r \leq N. \tag{14}$$

The denominator is

$$\begin{aligned} \sum_{i=0}^{r} p'_{ir} &= \sum_{i=0}^{r} [W(r-i+1) - W(r-i)] \\ &= W(r+1) - W(0) \\ &= W(r+1), \end{aligned} \tag{15}$$

such that

$$p_{nr} = \frac{W(r-n+1) - W(r-n)}{W(r+1)}, \quad n, r \leq N. \tag{16}$$

For $N = 5$ we have the matrix of p'_{nr} values:

$$(p_{nr}) = \begin{pmatrix} p_{00} & p_{01} & p_{02} & p_{03} & p_{04} & p_{05} \\ 0 & p_{11} & p_{12} & p_{13} & p_{14} & p_{15} \\ 0 & 0 & p_{22} & p_{23} & p_{24} & p_{25} \\ 0 & 0 & 0 & p_{33} & p_{34} & p_{35} \\ 0 & 0 & 0 & 0 & p_{44} & p_{45} \\ 0 & 0 & 0 & 0 & 0 & p_{55} \end{pmatrix}, \tag{17}$$

which is an upper triangular matrix of size 6×6, i.e. $(N+1) \times (N+1)$. With an extra day of data (setting $N = 6$) the matrix has an extra row (people with onset of symptoms on day 6 can now

be reported) and an extra column (the reported values on day 6):

$$(p_{nr}) = \begin{pmatrix} p_{00} & p_{01} & p_{02} & p_{03} & p_{04} & p_{05} & p_{06} \\ 0 & p_{11} & p_{12} & p_{13} & p_{14} & p_{15} & p_{16} \\ 0 & 0 & p_{22} & p_{23} & p_{24} & p_{25} & p_{26} \\ 0 & 0 & 0 & p_{33} & p_{34} & p_{35} & p_{36} \\ 0 & 0 & 0 & 0 & p_{44} & p_{45} & p_{46} \\ 0 & 0 & 0 & 0 & 0 & p_{55} & p_{56} \\ 0 & 0 & 0 & 0 & 0 & 0 & p_{66} \end{pmatrix}. \tag{18}$$

Given the data h_{nr} and these probabilities, we develop a multinomial log-likelihood function [17], adapting the approach of [18], to estimate the parameters λ and k. The log-likelihood function for λ and k, given the counts $\{h_{nr}\}$, is

$$l(\lambda, k | \{h_{nr}\}) = \log \left[\prod_{n=0}^{N} \prod_{r=n}^{N} p_{nr}^{h_{nr}} \right] \tag{19}$$

$$= \sum_{n=0}^{N} \sum_{r=n}^{N} h_{nr} \log p_{nr} \tag{20}$$

$$= \sum_{n=0}^{N} \sum_{r=n}^{N} h_{nr} \left\{ \log \left[W(r-n+1) - W(r-n) \right] - \log W(r+1) \right\}, \tag{21}$$

where the $W(\cdot)$ terms depend on λ and k. This is maximised numerically to give maximum likelihood estimates λ_{MLE} and k_{MLE} that are used in the differential equation model.

A key point to realise is that the definitions of p_{nr} for $r = 0$ to 5 (columns 1–6) are the same in (17) and (18), but their values will change from (17) to (18) because fitting the extra data from day 6 will update λ_{MLE} and k_{MLE}, which affects all the estimated values of the probabilities. Also, not all of the daily reported cases used in the differential equation model have details regarding onset of symptoms, i.e. $\sum_{n=0}^{N} h_{nr} \leq C_r$. Using the available data we obtain estimates (with 95% univariate confidence intervals) of $\lambda_{\text{MLE}} = 9.85$ $(9.30 - 10.46)$ and $k_{\text{MLE}} = 1.73$ $(1.60 - 1.86)$, as shown in Figure B. Using the maximum likelihood estimates, the mean delay is 8.78 days. This is one day longer than the data's raw mean (7.79 days) due to the right truncation (Figure B). These values were estimated on April 11 2020 using the 535 cases between February 29 and April 2, and used to parameterise the differential equation model. Due to a lag in the data being compiled, as of May 11 2020 there are now 667 cases between February 29 and April 2 that have dates of symptom onset. Note that these are what are shown in Figure B, with the fit using $\lambda_{\text{MLE}} = 9.84$ and $k_{\text{MLE}} = 1.75$ (which give a mean delay of 8.77 days). These are essentially unchanged (differences of ≤ 0.02) from the values estimated on April 11 that we used in the differential equation model.

For the New Zealand data on delays between symptom onset and reporting of cases (which are not publicly available), A. Lustig and M. Plank (pers. comm.) fit the delay distribution to NZ case reporting data using our R package. The resulting estimates are $\lambda_{\text{MLE}} = 7.82$ and $k_{\text{MLE}} = 1.53$, giving a mean delay of 7.04 days, which is 1.73 days shorter than for the BC data.

In the differential equation model, time is continuous and defined such that C_r, the number of recorded cases on day r, is equal to the number of cases recorded by the end of day r. The number

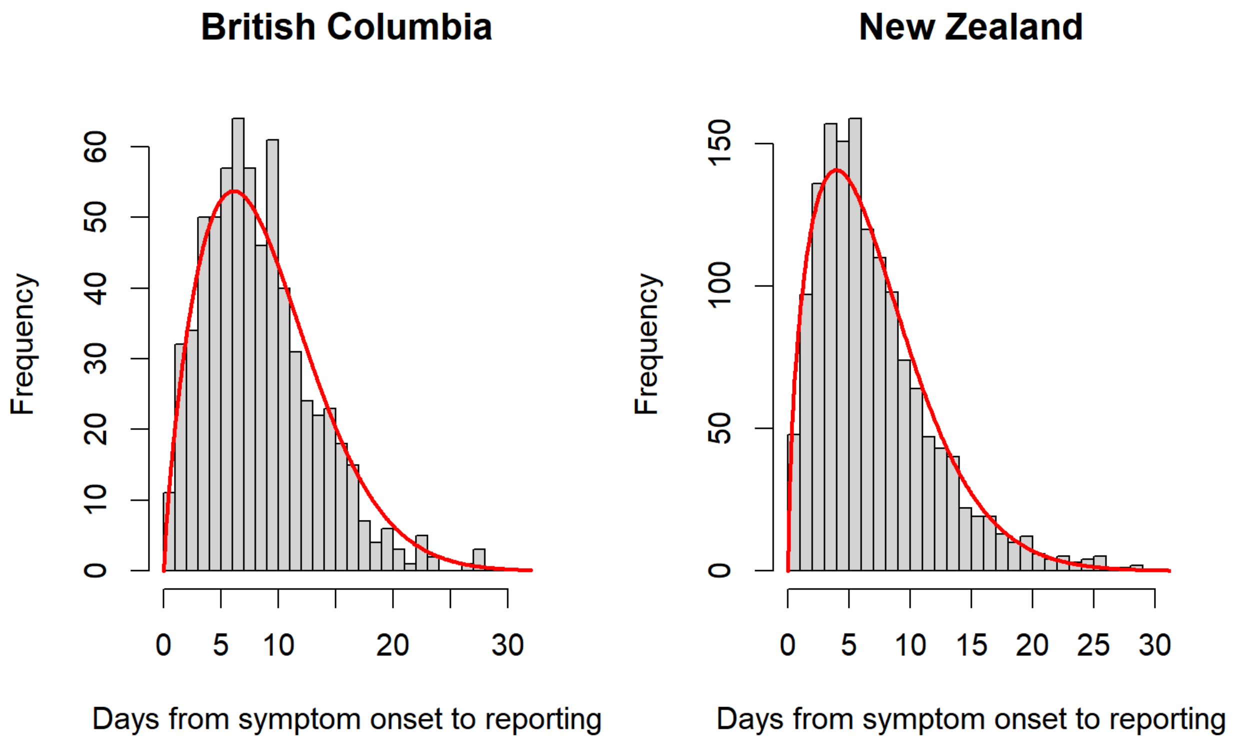

Figure B: Histograms of delays between onset of symptoms and a case being reported, from the case-specific data for British Columbia (left) and New Zealand (right). The red lines show the maximum likelihood fit of the Weibull distribution, taking into account the right-truncation of the data. For British Columbia, the distribution estimates longer delays (mean of 8.78 days) than shown in the data (mean of 7.79 days) due to the fit accounting for right-truncation—there has not yet been the opportunity to record many long delays (Fig 1).

of people per day who become symptomatic throughout day n is the total number moving from the E_2 and $E_{2\mathrm{d}}$ compartments to the I and I_d compartments, which is

$$\int_{n-1}^{n} k_2 \left[E_2(\tau) + E_{2\mathrm{d}}(\tau) \right] \mathrm{d}\tau. \tag{22}$$

The expected number of cases that are reported on day r is then

$$\mu_r = \psi_r \sum_{n=1}^{r} \int_{n-1}^{n} k_2 \left[E_2(\tau) + E_{2\mathrm{d}}(\tau) \right] w(r-\tau) \mathrm{d}\tau \tag{23}$$

$$= \psi_r \int_{0}^{r} k_2 \left[E_2(\tau) + E_{2\mathrm{d}}(\tau) \right] w(r-\tau) \mathrm{d}\tau, \tag{24}$$

where ψ_r represents the sampling fraction on day r and $w(\cdot)$ represents the Weibull distribution with parameters λ_{MLE} and k_{MLE}. If $\psi_r = 1$ then all estimated infectious people are tested and then become reported cases; $\psi_r < 1$ represents a reduction in expected cases on day r due to not

everyone being tested. By changing variables $\tau = r - s$, this becomes

$$\begin{aligned} \mu_r &= -\psi_r \int_r^0 k_2 \left[E_2(r-s) + E_{2\mathrm{d}}(r-s) \right] w(s) \mathrm{d}s \qquad (25) \\ &= \psi_r \int_0^r k_2 \left[E_2(r-s) + E_{2\mathrm{d}}(r-s) \right] w(s) \mathrm{d}s. \qquad (26) \end{aligned}$$

The differential equation model is started from a time earlier than $r = 0$, so the integration here can go back further because the model calculates, for example, $E_2(-1)$; $r < 0$ does not occur in the above likelihood calculations because, by definition, there are no data for such times. This requires setting some maximum delay between symptom onset and a case being reported that is large enough for $w(s)$ to be negligible. Based on Figure B we use a maximum delay of 45 days (the 99.99992% quantile), giving

$$\mu_r = \psi_r \int_0^{45} k_2 \left[E_2(r-s) + E_{2\mathrm{d}}(r-s) \right] w(s) \mathrm{d}s, \qquad (27)$$

which is calculated and compared to the data (C_r) via the negative binomial observation model.

Model Validation

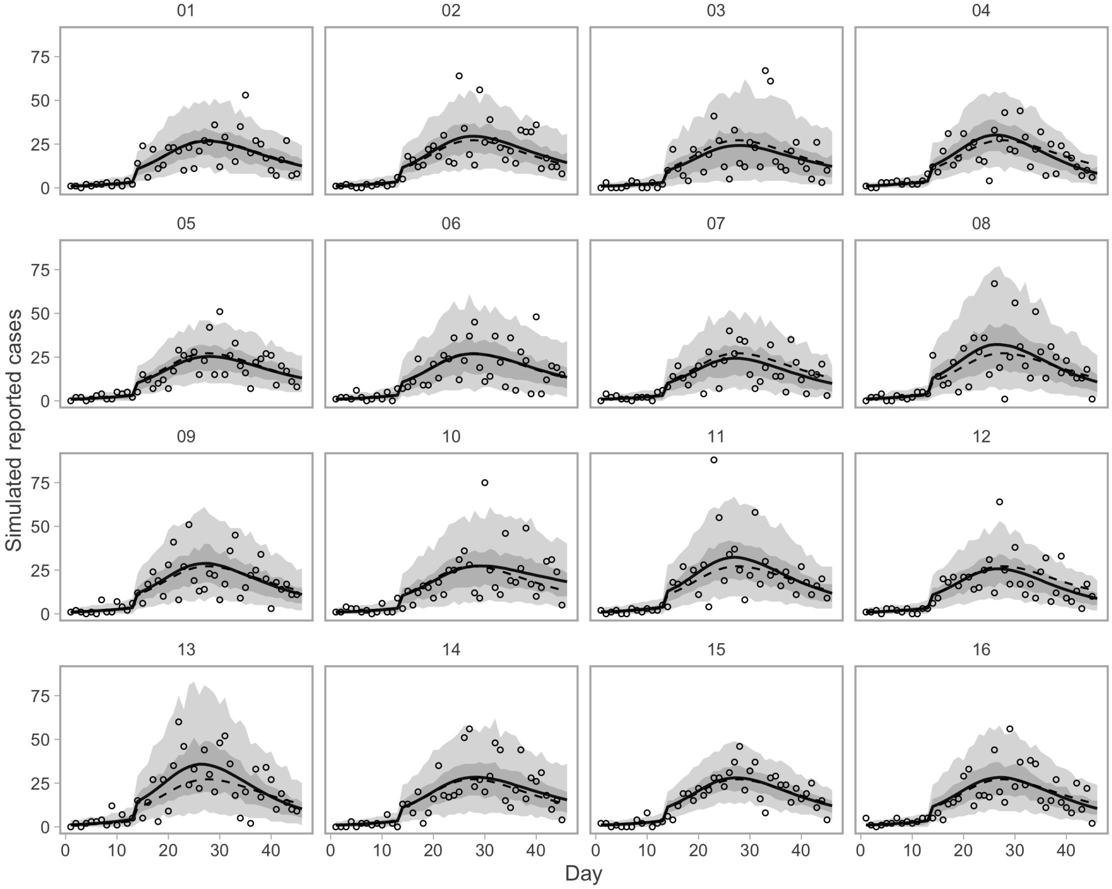

Figure C: Simulation testing the ability to recover time series of reported cases. Dashed lines represent the true simulated time series. Solid lines represent means of the posterior. Dots represent observed simulated data. Shaded ribbons represent 50% and 90% credible intervals on new observations and should ideally encompass 50% and 90% of the simulated data points, respectively.

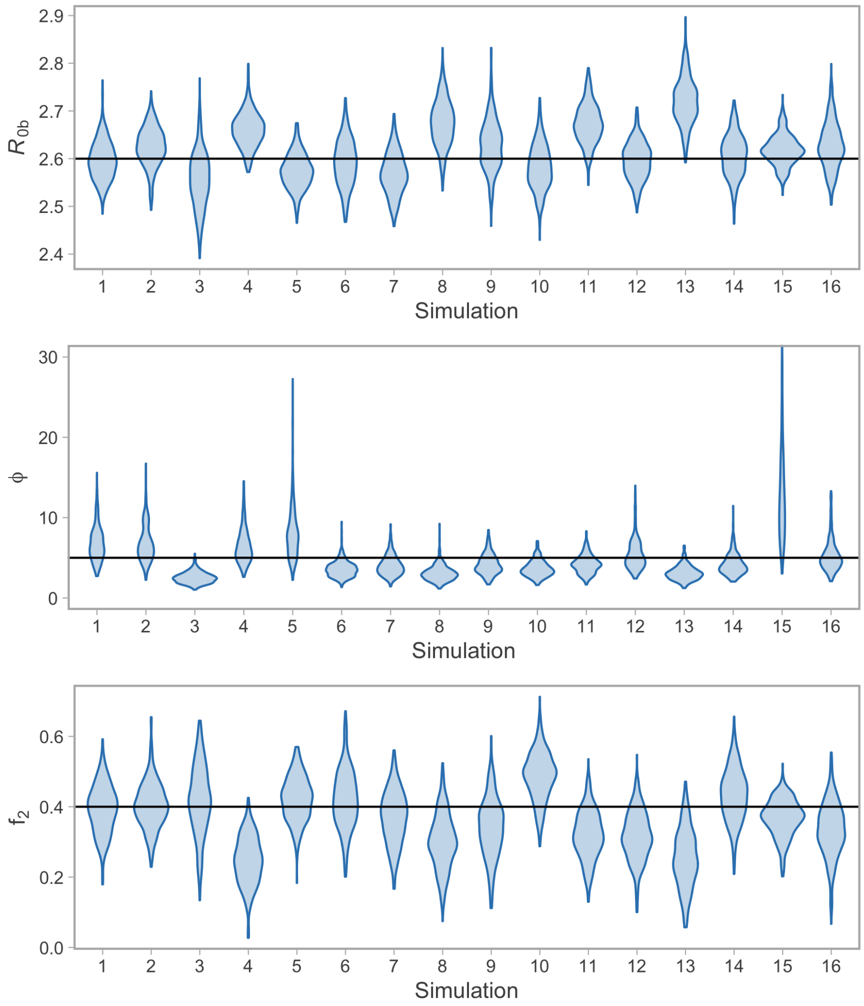

Figure D: Simulation testing the ability to recover parameter values. The parameter R_{0b} is the basic reproductive number of the model without interventions. The parameter ϕ is the (inverse) dispersion parameter of the negative binomial observation model. The fraction of normal contacts is f_2. The “violins” illustrate the posterior density distribution across 16 simulation examples. The true values used in the simulation are indicated by the horizontal black lines.

Supplemental Results

Table C: Posterior means, quantiles, effective sample sizes (ESS), and potential scale reduction factors ($\hat{R}$) for the main model fit (Fig. 3). The chains are consistent with convergence as $\hat{R}$ approaches 1.

Parameter	Mean	2.5%	50%	97.5%	ESS	$\hat{R}$
R_{0b}	2.95	2.88	2.95	3.02	2685.3	1.002
f_2	0.22	0.08	0.22	0.36	2697.0	1.001
ϕ	6.86	3.39	6.52	12.37	3410.0	1.002

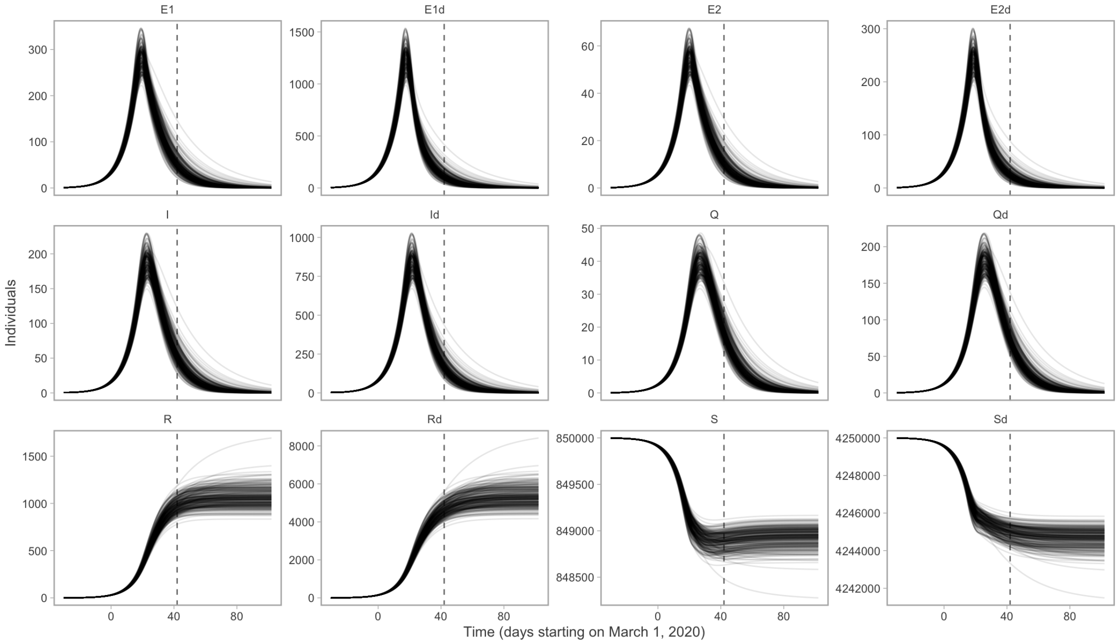

Figure E: Posterior distributions of the underlying state variables. The time-dependent variables are the numbers of individuals who are: susceptible (S), exposed to the virus, asymptomatic and not infectious (E_1), exposed, asymptomatic and infectious (E_2), infectious (I), quarantined (Q), and recovered and deceased (R). Recovered individuals are assumed to be immune. There are analogous variables for individuals practising physical distancing, represented by subscript d, i.e., S_d, $E_{1\mathrm{d}}$, $E_{2\mathrm{d}}$, I_d, Q_d, and R_d.

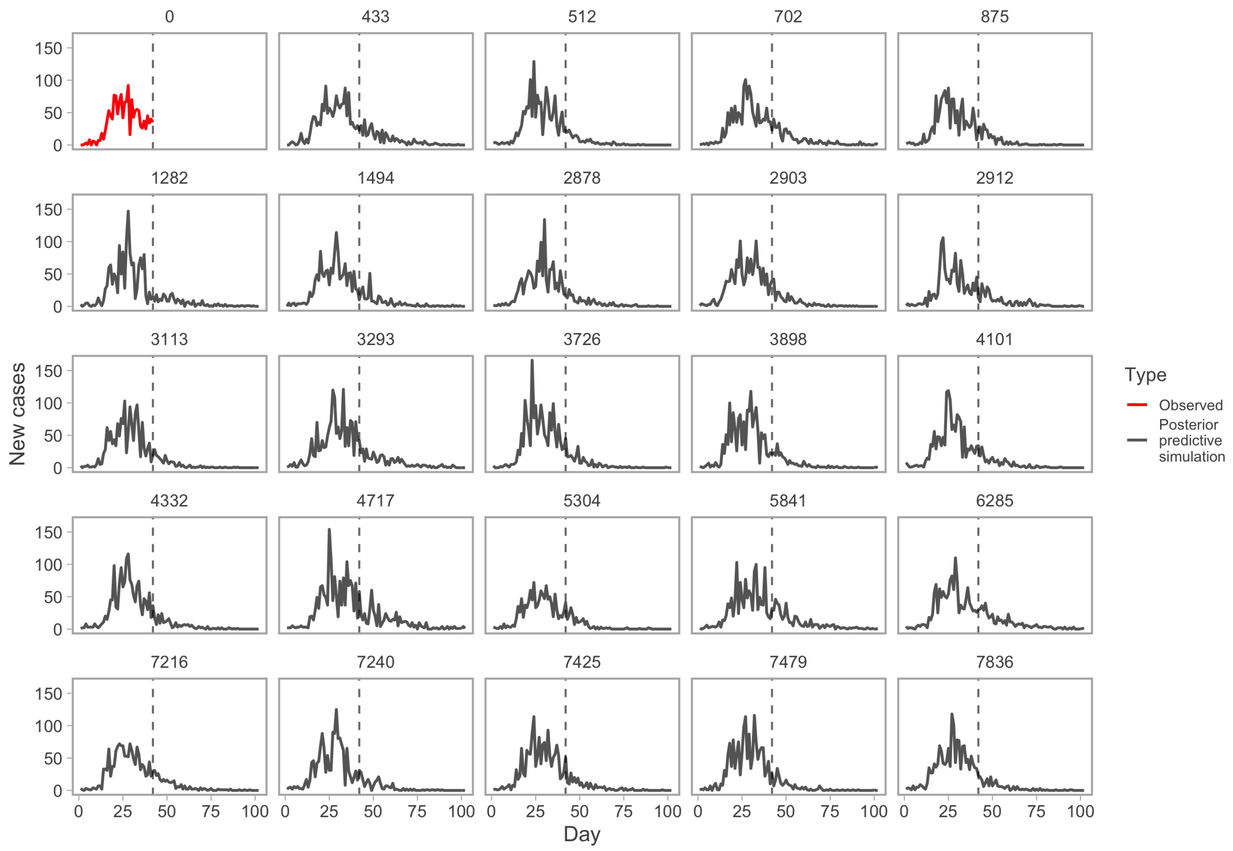

Figure F: Example posterior-predictive replicates from the model fit. The first panel (red line) represents the observed data. All other panels represent example draws from the posterior-predictive distribution. Numbers above panels represent randomly selected Markov chain Monte Carlo (MCMC) iterations. This plot helps evaluate whether the observed data are consistent with data generated by the model.

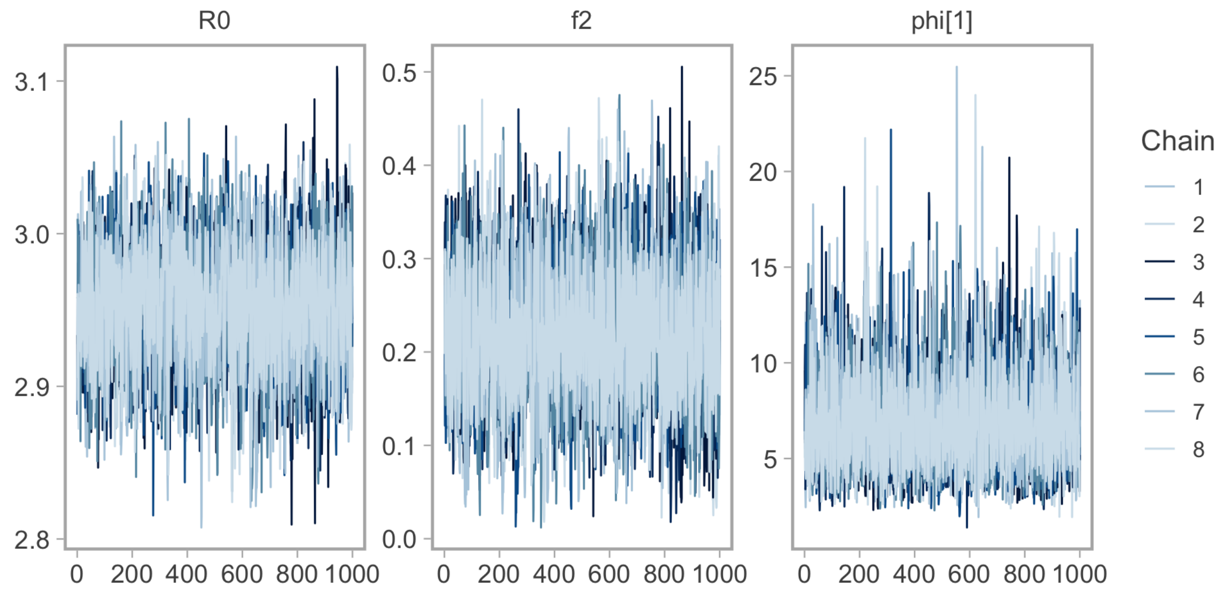

Figure G: Trace plots of MCMC (Markov chain Monte Carlo) samples from parameter distributions to assess chain convergence. The panel labelled "R0" actually refers to R_{0b}. The panel labelled "phi[1]" refers to the (inverse) dispersion parameter ϕ from the negative binomial observation model.

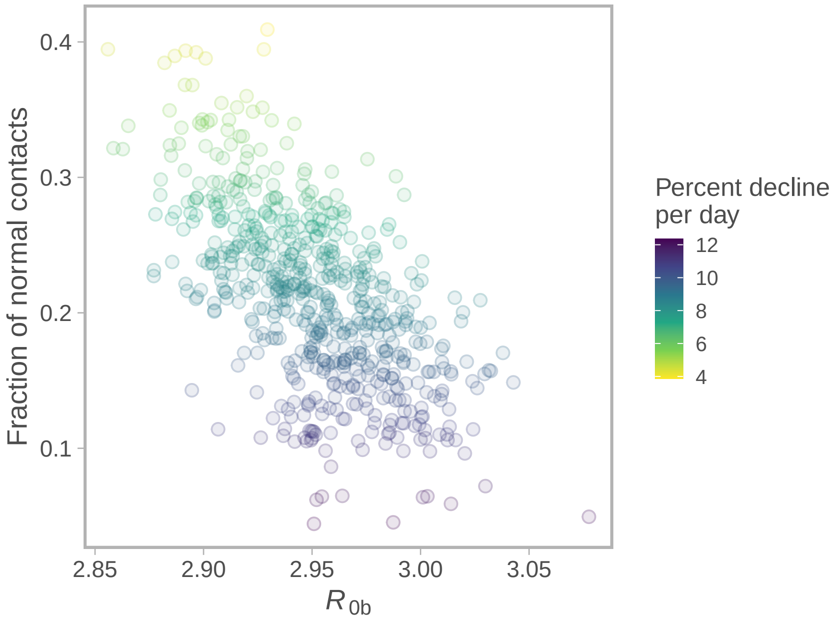

Figure H: Joint posterior of fraction normal contacts vs. R_{0b}. Colour represents percent decline per day over the last 30 days of the projection. Dots represent individual draws from the joint posterior.

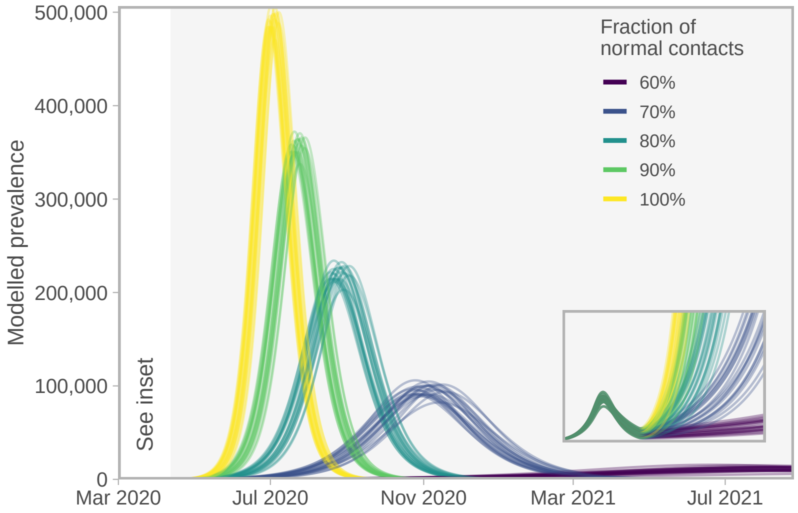

Figure I: Epidemiological curves for five scenarios of relaxing distancing measures (beginning on April 12 2020) that are insufficient to keep the prevalence from growing. Stronger measures "flatten the curve", as would be expected. Lines represent 20 draws from the joint posterior distribution. Grey region denotes future dates during which the relaxed distancing measures are applied. Inset box magnifies the lower-left region of the plot to show the modelled prevalence from March 1 to July 1 2020—the initial curve is imperceptible on the main axes. **Note**: Model prevalence depends on our assumptions about underestimation, incubation period, and the duration of infection, none of which we can estimate well from these data (Figure M). This model does not include individuals who do not develop symptoms. Much higher values of the prevalence are consistent with our data.

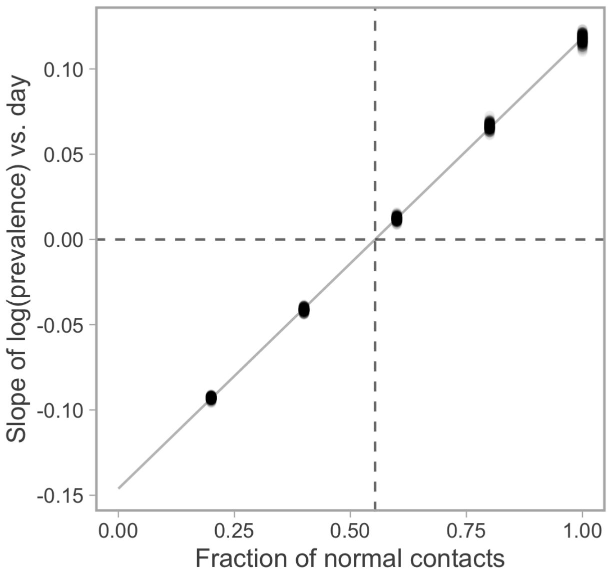

Figure J: Linear regression of [the slope of log(prevalence) over days (for the last 30 days of the projection)] vs. [fraction of normal contacts]. Vertical dashed line indicates the critical value below which the rate of change of prevalence becomes positive. Dots represent individual posterior draws.

Table D: Posterior means, quantiles, effective sample sizes (ESS), and potential scale reduction factors ($\hat{R}$) for model fits to regions other than BC (Fig. 5). The chains are consistent with convergence as $\hat{R}$ approaches 1. The parameters "f_2 ramp start" and "f_2 ramp end" refer to the estimated days since the starting date of the data (Table B) that the physical distancing contact fraction (f_2) begins and finishes being linearly "ramped in" from the initial R_{0b}. These ramp date parameters were not estimated in the main BC model fit.

Region	Parameter	Mean	2.5%	50%	97.5%	ESS	$\hat{R}$
New York	R_{0b}	6.57	6.42	6.57	6.72	772	1.01
New York	f_2	0.18	0.12	0.18	0.23	497	1.00
New York	ϕ	4.37	2.99	4.33	6.14	602	1.01
New York	f_2 ramp start	11.69	8.26	11.65	15.04	546	1.00
New York	f_2 ramp end	25.48	20.90	25.37	30.70	476	1.00
Florida	R_{0b}	4.74	4.64	4.73	4.85	1401	1.00
Florida	f_2	0.32	0.28	0.32	0.37	1325	1.00
Florida	ϕ	5.55	3.47	5.41	8.34	1515	1.00
Florida	f_2 ramp start	13.67	11.21	13.67	16.26	1394	1.00
Florida	f_2 ramp end	27.50	24.21	27.47	30.82	1141	1.00
Washington	R_{0b}	5.02	4.93	5.02	5.13	1407	1.00
Washington	f_2	0.30	0.28	0.30	0.32	1297	1.00
Washington	ϕ	10.68	6.91	10.52	15.53	1304	1.00
Washington	f_2 ramp start	8.58	7.21	8.53	10.21	1542	1.00
Washington	f_2 ramp end	18.63	16.21	18.64	21.14	1378	1.00
New Zealand	R_{0b}	8.98	8.74	8.98	9.22	847	1.01
New Zealand	f_2	0.07	0.03	0.07	0.11	742	1.00
New Zealand	ϕ	11.10	3.85	8.98	30.98	789	1.00
New Zealand	f_2 ramp start	3.19	2.65	3.17	3.83	1141	1.00
New Zealand	f_2 ramp end	12.31	10.62	12.30	14.00	736	1.01
California	R_{0b}	5.08	4.97	5.07	5.19	2430	1.00
California	f_2	0.41	0.38	0.41	0.44	2176	1.00
California	ϕ	8.51	5.46	8.36	12.24	3051	1.00
California	f_2 ramp start	7.75	6.25	7.72	9.41	2868	1.00
California	f_2 ramp end	24.66	21.28	24.71	27.93	1998	1.00

Sensitivity Analysis

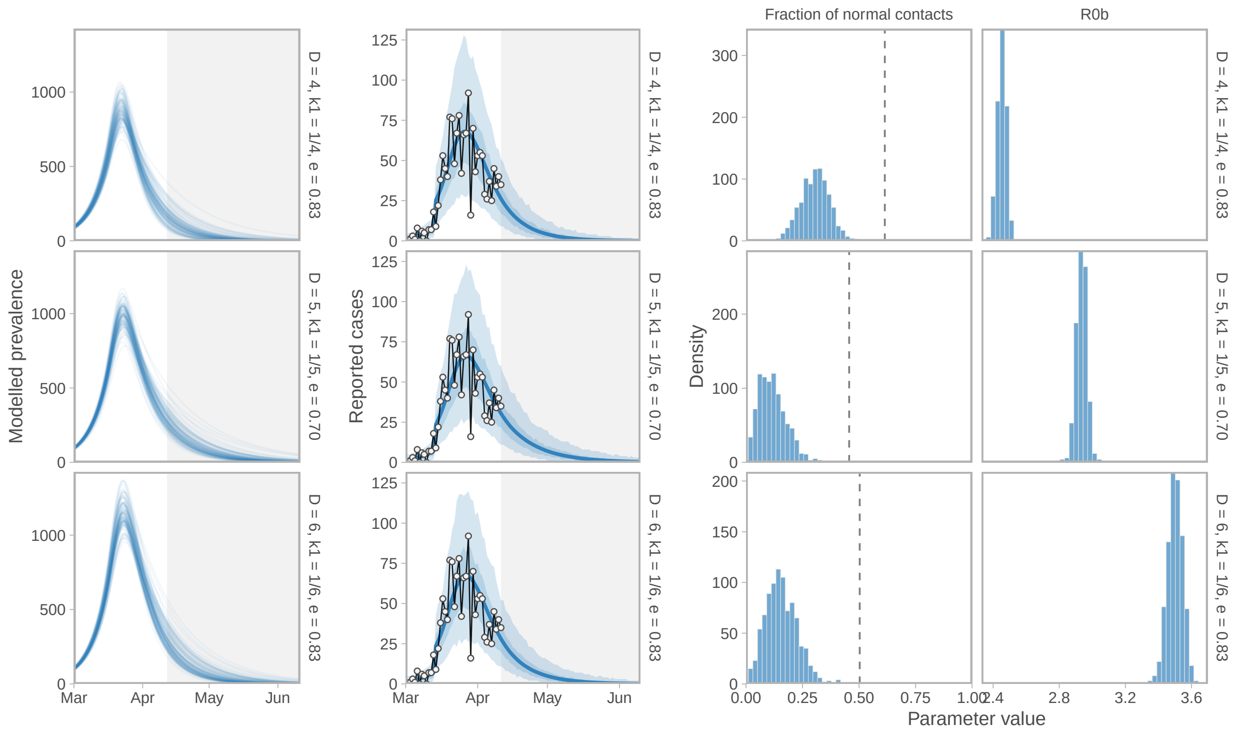

Figure K: Sensitivity of prevalence and case-count projections, R_{0b}, and the fraction-of-normal-contacts estimate (f_2) to three sets of alternative parameter values: (1) $D = 4$, $k_1 = 1/4$, $u_r = 0.2$ (shorter duration); (2) $D = 5$, $k_1 = 1/5$, $u_r = 0.3/0.7$ (a lower proportion of people physical distancing: 70% vs. 83% in the main analysis); and (3) $D = 6$, $k_1 = 1/6$, $u_r = 0.2$ (longer duration). Histograms illustrate the posterior distribution of MCMC samples. Dashed vertical line represents the threshold value below which an exponential increase occurs.

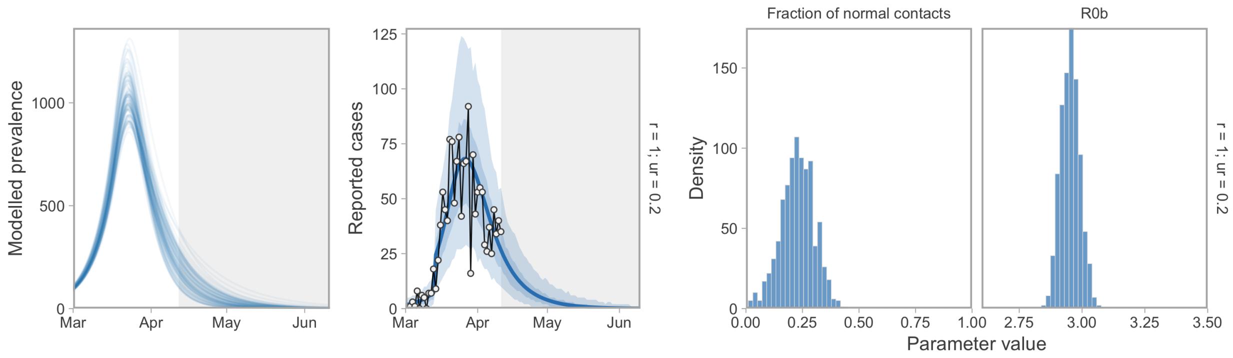

Figure L: Prevalence and case-count projections, R_{0b}, and the fraction-of-normal-contacts estimate (f_2) with distancing-rate parameters $u_d = 1$ and $u_r = 0.2$. The ratio e is the same as at baseline but these rates are 10 times higher. The model dynamics are sensitive to e but not to the choice of rates. Accordingly, these estimates are as in Fig 3.

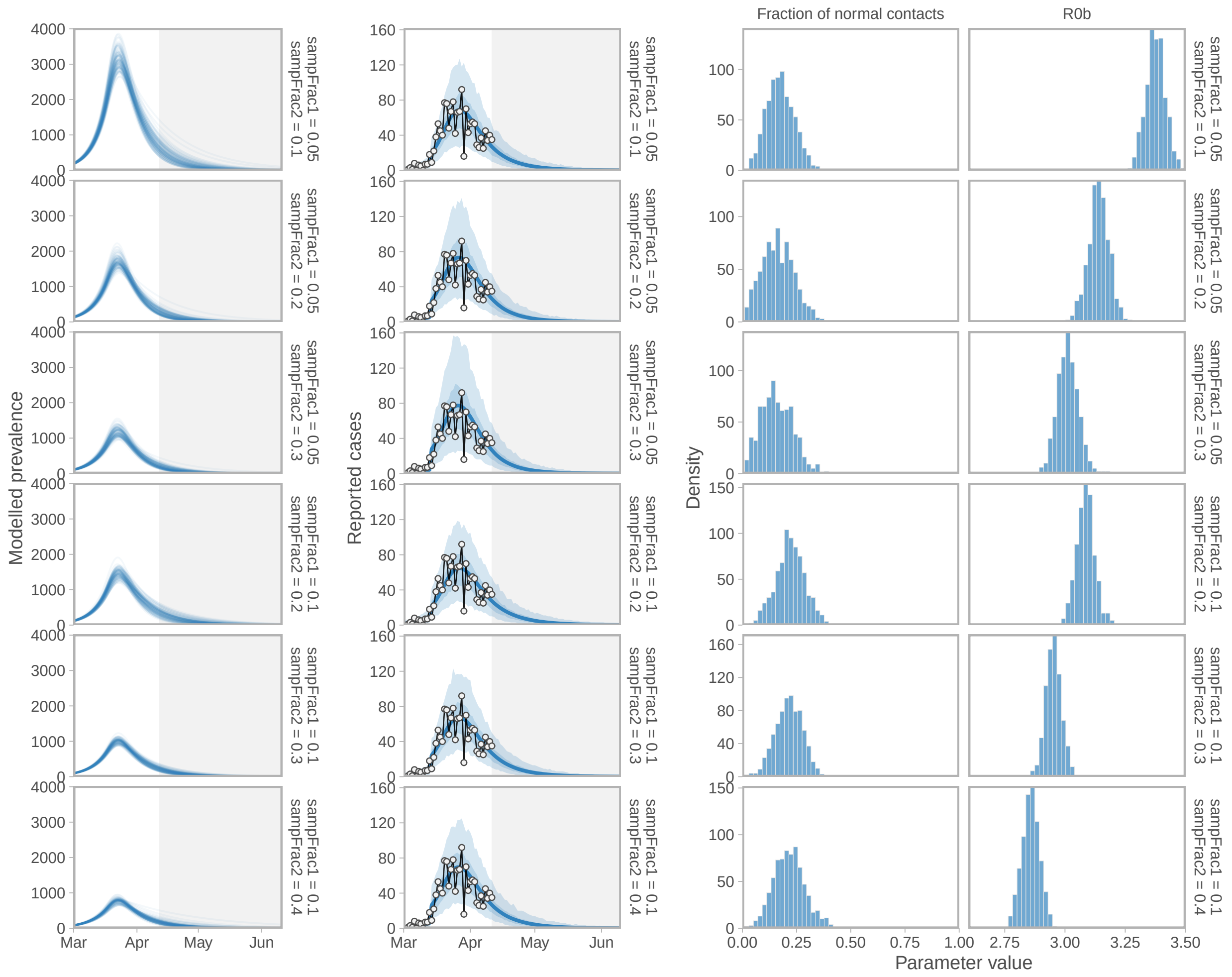

Figure M: Sensitivity of prevalence and case-count projections, R_{0b}, and the fraction of normal contacts (f_2) to assumed fractions of positive cases sampled. "sampFrac1" refers to the assumed sample fraction before March 14, 2020 and "sampFrac2" refers to the assumed sample fraction on and after March 14, 2020. Histograms illustrate the posterior distribution of MCMC samples.

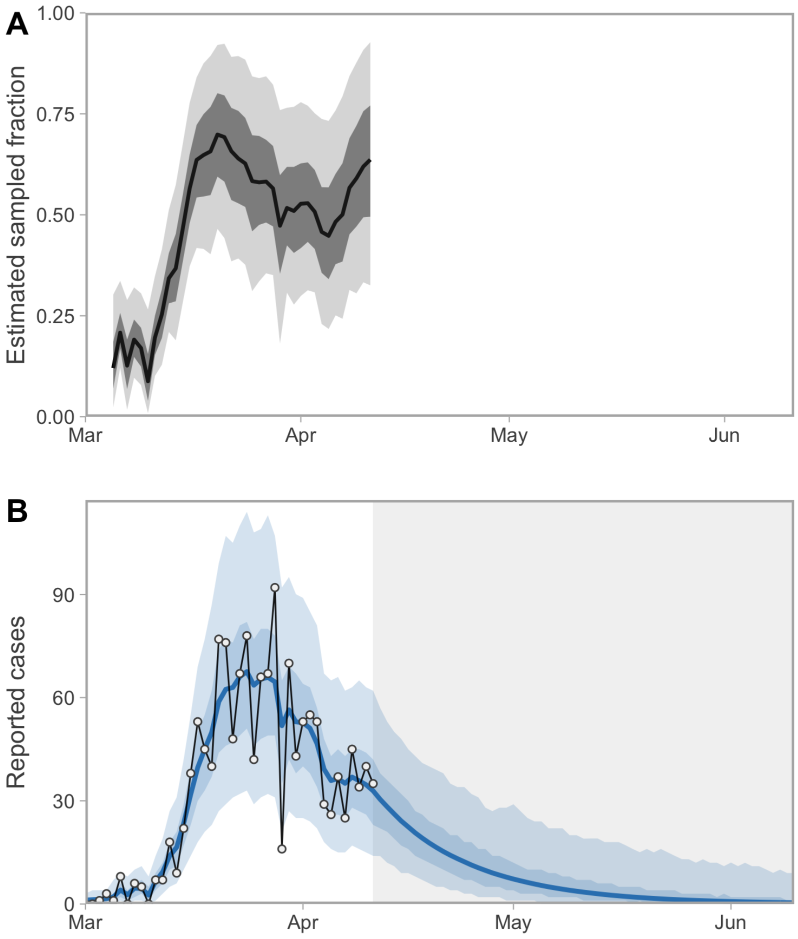

Figure N: Alternative model fitted with a random walk on the fraction of positive cases sampled. (A) Estimated sampled fraction random walk. The random walk starts on the fifth day (it is fixed at 0.1 before then) and then evolves with a fixed standard deviation of 0.1. We placed a Beta prior on the initial value with a mean and standard deviation of 0.2 and 0.2. (B) Resulting model fit with a sampled-fraction random walk. Shaded ribbons represent 50% and 90% credible intervals. Thick lines represent medians (A) and means (B) of the posterior distribution. Although the shape in panel A is plausible, we deemed the scale of the estimated values to be implausibly high and therefore used a fixed value of 0.3 after day 14 in the main models in this paper.

References

1. Hilbe JM. Negative Binomial Regression. Cambridge University Press; 2011.

2. MIDAS Network. COVID-19;. https://github.com/midas-network/COVID-19.

3. Liu Q, Liu Z, Zhu J, Zhu Y, Li D, Gao Z, et al. Assessing the Global Tendency of COVID-19 Outbreak. medRxiv. 2020;doi:10.1101/2020.03.18.20038224.

4. Stan Development Team. Prior Choice Recommendations; 2020. Available from: https://github.com/stan-dev/stan/wiki/Prior-Choice-Recommendations.

5. Korzinski D, Kurl S. COVID-19 Carelessness: Which Canadians say pandemic threat is 'overblown'? And how are they behaving in turn?; 2020. Available from: http://angusreid.org/covid-19-serious-vs-overblown/.

6. Russell TW, Hellewell J, Abbott S, Golding N, Gibbs H, Jarvis CI, et al.. Using a delay-adjusted case fatality ratio to estimate under-reporting; 2020. https://cmmid.github.io/topics/covid19/global_cfr_estimates.html.

7. Gelman A, Carlin JB, Stern HS, Dunson DB, Vehtari A, Rubin DB. Bayesian Data Analysis. 3rd ed. Boca Raton, FL: Chapman & Hall; 2014.

8. Google. COVID-19 Community Mobility Reports; 2020. https://www.google.com/covid19/mobility.

9. The COVID Tracking Project;. https://covidtracking.com/.

10. New Zealand Ministry of Health. COVID-19—current cases details; 2020. https://www.health.govt.nz/our-work/diseases-and-conditions/covid-19-novel-coronavirus/covid-19-current-situation/covid-19-current-cases/covid-19-current-cases-details.

11. Diekmann O, Heesterbeek JAP, Metz JA. On the definition and the computation of the basic reproduction ratio R_0 in models for infectious diseases in heterogeneous populations. J Math Biol. 1990;28(4):365–382.

12. van den Driessche P, Watmough J. Reproduction numbers and sub-threshold endemic equilibria for compartmental models of disease transmission. Math Biosci. 2002;180:29–48. doi:10.1016/S0025-5564(02)00108-6.

13. Stein WA, et al.. Sage Mathematics Software (Version 9.0); 2020. Available from: http://www.sagemath.org.

14. SageMath I. CoCalc Collaborative Computation Online; 2016. Available from: https://cocalc.com.

15. Edwards AM. rightTruncation: R package for maximum likelihood estimation of delay distributions for right-truncated data (as during COVID-19); 2020. Available from: https://github.com/andrew-edwards/rightTruncation.

16. Backer JA, Klinkenberg D, Wallinga J. Incubation period of 2019 novel coronavirus (2019-nCoV) infections among travellers from Wuhan, China, 20-28 January 2020. Euro Surveill. 2020;25:2000062. doi:10.2807/1560-7917.ES.2020.25.5.2000062.

17. Lawless JF. Statistical Models and Methods for Lifetime Data. New Jersey: 2nd ed., Wiley series in Probability and Statistics, Wiley; 2003.

18. Edwards AM, Robinson JPW, Blanchard JL, Baum JK, Plank MJ. Accounting for the bin structure of data removes bias when fitting size spectra. Mar Ecol Prog Ser. 2020;636:19–33. doi:10.3354/meps13230.
